# Feasibility of Azacitidine Added to Standard Chemotherapy in Older Patients with Acute Myeloid Leukemia — A Randomised SAL Pilot Study

**DOI:** 10.1371/journal.pone.0052695

**Published:** 2012-12-31

**Authors:** Utz Krug, Anja Koschmieder, Daniela Schwammbach, Joachim Gerss, Nicola Tidow, Björn Steffen, Gesine Bug, Christian H. Brandts, Markus Schaich, Christoph Röllig, Christian Thiede, Richard Noppeney, Matthias Stelljes, Thomas Büchner, Steffen Koschmieder, Ulrich Dührsen, Hubert Serve, Gerhard Ehninger, Wolfgang E. Berdel, Carsten Müller-Tidow

**Affiliations:** 1 Department of Medicine A, University Hospital, Muenster, Germany; 2 Institute of Biostatistics and Clinical Research, University Hospital, Muenster, Germany; 3 Department of Medicine, Hematology/Oncology, Goethe University, Frankfurt am Main, Germany; 4 Department of Medicine I, University of Technics, Dresden, Germany; 5 Department of Hematology, University Hospital, Essen, Germany; Cardiff University, United Kingdom

## Abstract

**Introduction:**

Older patients with acute myeloid leukemia (AML) experience short survival despite intensive chemotherapy. Azacitidine has promising activity in patients with low proliferating AML. The aim of this dose-finding part of this trial was to evaluate feasibility and safety of azacitidine combined with a cytarabine- and daunorubicin-based chemotherapy in older patients with AML.

**Trial Design:**

Prospective, randomised, open, phase II trial with parallel group design and fixed sample size.

**Patients and Methods:**

Patients aged 61 years or older, with untreated acute myeloid leukemia with a leukocyte count of <20,000/µl at the time of study entry and adequate organ function were eligible. Patients were randomised to receive azacitidine either 37.5 (dose level 1) or 75 mg/sqm (dose level 2) for five days before each cycle of induction (7+3 cytarabine plus daunorubicine) and consolidation (intermediate-dose cytarabine) therapy. Dose-limiting toxicity was the primary endpoint.

**Results:**

Six patients each were randomised into each dose level and evaluable for analysis. No dose-limiting toxicity occurred in either dose level. Nine serious adverse events occurred in five patients (three in the 37.5 mg, two in the 75 mg arm) with two fatal outcomes. Two patients at the 37.5 mg/sqm dose level and four patients at the 75 mg/sqm level achieved a complete remission after induction therapy. Median overall survival was 266 days and median event-free survival 215 days after a median follow up of 616 days.

**Conclusions:**

The combination of azacitidine 75 mg/sqm with standard induction therapy is feasible in older patients with AML and was selected as an investigational arm in the randomised controlled part of this phase-II study, which is currently halted due to an increased cardiac toxicity observed in the experimental arm.

**Trial Registration:**

This trial is registered at clinical trials.gov (identifier: NCT00915252).

## Introduction

Despite intensive treatment, acute myeloid leukemia (AML) in elderly patients still has a dismal outcome with the vast majority of patients succumbing to their disease within 2 years after diagnosis [1]. Intrinsic biological differences lead to a complete remission rate of only about 50% compared to approximately 70% in elderly patients [1], [2]. Aberrant DNA methylation patterns can frequently be detected in AML blasts [3], and gene mutations that alter DNA methylation patterns were recently identified in AML, among them mutations of the isocitrate dehydrogenase genes (IDH) 1 and 2 [4] and DNA methyltransferase 3A [5]. The hypomethylating agents azacitidine and decitabine are able to reverse aberrant promoter hypermethylation by inhibition of DNA methyltransferases [6]. Clinical activity with prolongation of survival compared to conventional care was demonstrated for azacitidine in patients with high-risk myelodysplastic syndrome (MDS) and low proliferating AML with up to 30% bone marrow blasts [7], [8] and ongoing trials currently evaluate the efficacy of azacitidine and decitabine in AML with >30% bone marrow blasts. However, despite their clinical activity, these compounds do not lead to long-term remissions.

*In vitro* data suggest a synergistic effect of cytarabine and azacitidine when azacitidine is administered before cytarabine exposure [9]. This might in part be explained by the induction of deoxycytidine kinase (dCK) by azacitidine. dCK phosphorylates cytarabine to its active compound, ara-CTP [10].

While high-dose cytarabine has demonstrated strong clinical activity [11], the failure of chemotherapy to cure a patient of AML is often due to cellular cytarabine resistance [12]. Several potential resistance mechanisms are discussed, among those an inactivity of dCK. In a cytarabine-resistent, dCK-deficient leukemic cell line originating from HL60, sensitivity towards cytarabine could be restored upon induction of dCK by azacitidine [10].

Even though no inactivation of dCK could be demonstrated *in vivo* in cytarabine resistant primary AML cells, a phase I-study with 17 pediatric patients with relapsed acute lymphoblastic leukemia (ALL) after high-dose cytarabine treatment yielded a complete remission in two out of 9 evaluable patients after treatment with azacitidine followed by another course of high-dose cytarabine [13].

The aim of this pilot trial was to establish the preliminary safety profile of azacitidine added to standard induction and consolidation therapy in older patients with newly diagnosed AML, and to determine the recommended dose for a subsequent controlled phase II trial.

## Patients and Methods

### Study Design and Eligibility

The protocol for this trial and supporting CONSORT checklist are available as supporting information; see Checklist S1 and Protocol S1. The dose finding trial reported herein was a prospective, randomised, open-label phase II trial with parallel group design and fixed sample size preceeding an open-label, randomised, controlled, multicenter phase II trial of the study alliance leukemia (SAL). Participating centers of the dose finding part of this trial were the University Hospitals of Muenster, Frankfurt am Main, Dresden and Essen, all located in Germany. The trial was registered at www.clinicaltrials.gov (identifier: NCT00915252, URL: http://clinicaltrials.gov/ct2/show/NCT00915252). The study was approved by the Ethics Committee of the University of Muenster and conducted in accordance with the Declaration of Helsinki. Written, informed consent was obtained from all patients before study enrolment.

Patients with >60 years of age with untreated AML (excluding acute promyelocytic leukemia) were eligible. Exclusion criteria were: Known central nervous system manifestation of AML, heart failure class 3 or 4 according to New York Heart Association, unstable coronary artery disease, serious cardiac ventricular arrhythmias requiring anti-arrhythmic therapy beyond beta blockers or digoxin, inadequate renal (creatinin clearance <30 ml/min) or hepatic function (serum total bilirubin ≥1.5 upper limit of normal (ULN), alanine aminotransferase (ALT) or aspartate aminotransferase (AST) ≥2.5×ULN not caused by leukemic infiltration), known human immunodeficiency virus and/or hepatitis C infection, evidence or history of severe non-leukemia associated bleeding diathesis or coagulopathy, evidence or recent history of CNS disease, including primary or metastatic brain tumors, seizure disorders, uncontrolled active infection, concurrent malignancies other than AML with an estimated life expectancy of less than two years, history of organ allograft, hypersensitivity to cytarabine (not including drug fever or exanthema), daunorubicin, azacitidine or mannitol and previous therapy with azacitidine (i.e. for an antecedent myelodysplastic syndrome). An exclusion criterion only for the dose finding part of this trial was a leukocytosis >20,000/µl at study entry. For the controlled part of the trial, those patients were eligible if the leukocyte count could be decreased to <20,000/µl with hydroxyurea.

### Randomisation

Patients were randomised 1∶1 between 37.5 (dose level 1) and 75 mg/sqm (dose level 2) of azacitidine without stratification. This parallel randomised evaluation allowed us a rapid evaluation of two different dose levels of azacitidine, and ensured six patients being treated with the recommended dose for the controlled part of the study. The random allocation sequence was generated using SAS software (proc plan). Randomisation lists were generated using block randomisation with a block size of 4. Randomisation was performed centrally providing allocation concealment. Patients were first screened for eligibility at clinical sites without having access to randomisation lists. Eligible patients were reported to a central randomisation unit by fax. Subsequently the intended intervention of each patient was reported back to clinical investigators.

### Treatment Plan

**Figure 1:**
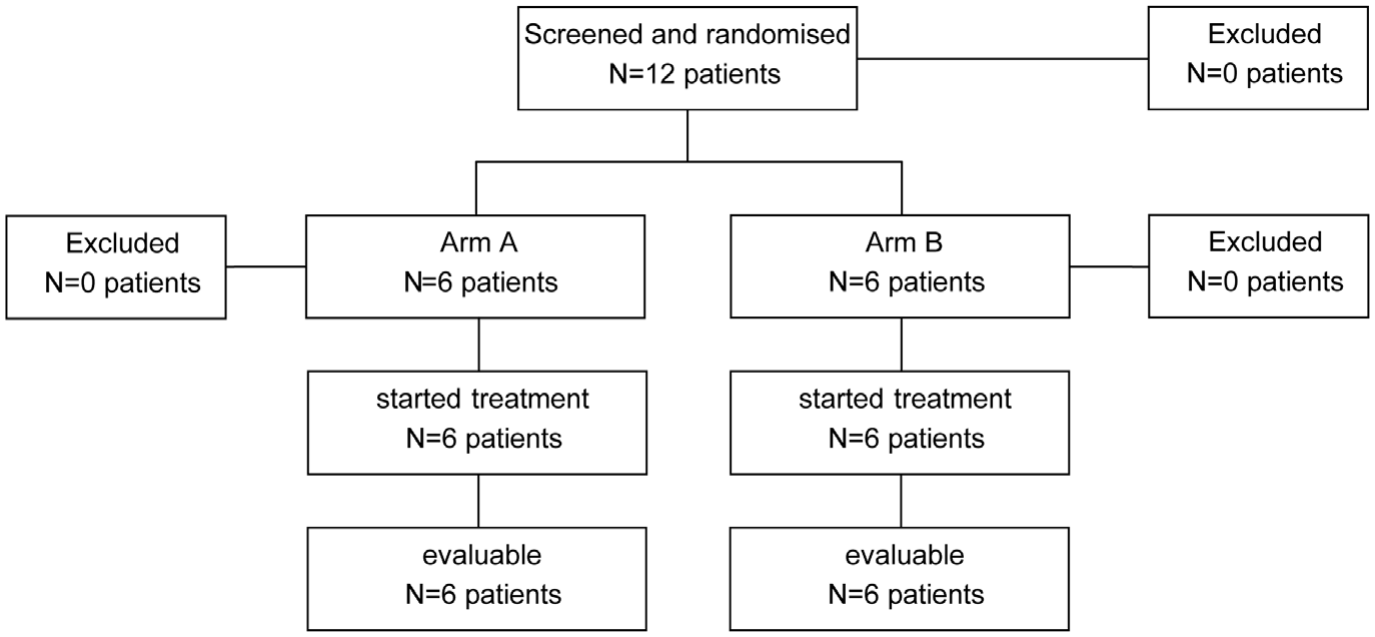
CONSORT statement. The patient flow through this dose finding pilot trial is shown.

The treatment scheme is depicted in Fig. 1. After randomisation, patients received the randomised dose level of azacitidine on days −5 to −1 as intravenous (iv) infusion over 30 minutes, followed by ‘7+3’ induction chemotherapy consisting of cytarabine 100 mg/sqm continuous iv infusion on days 1–7 and daunorubicin 45 mg/sqm iv on days 3–5. The rationale for reducing the azacitidine application per cycle to five days, as opposed to seven days per cycle for monotherapy, were as follows: First, the five-days application allowed for an application on weekdays, since application on weekends poses problems in selected study centers. Second, a pretreatment >5 days would prolong the delay of initiation of cytotoxic therapy in those patients with untreated AML. The maximum dose of 5×75 mg/sqm resulted from concerns regarding an additional cytotoxicity of the combination of standard induction and postremission therapy and higher doses of azacitidine. Dose level 1 of azacitidine was selected as 50% of dose level 2 based on recommended dose reductions to 50% in case of prolonged hematotoxicity by the manufacturer (investigational brochure and subsequent prescribing information).

An early bone marrow evaluation was performed seven days after the end of the first induction therapy. A sufficient blast clearance in the early bone marrow evaluation was defined as <5% remaining leukemic blasts according to the revised recommendations of the International Working Group for AML [14]. Only in case of ≥5% residual bone marrow blasts did patients receive an identical second induction course starting with azacitidine on day 17. Patients that achieved a complete remission (CR) after induction therapy received two consolidation courses of azacitidine at the randomised dose level on days −5 to −1 subcutaneously, followed by intermediate-dose cytarabine (1 g/sqm bidaily iv on days 1, 3 and 5). Stopping rules included an increase of the leukocyte count to >50.000/µl during the first five days of azacitidine treatment. Such a patient would immediately terminate azacitidine treatment and start standard induction therapy. A patient was evaluable if either 1. at least one therapy cycle with azacitidine plus induction therapy was completed or 2. patient received at least one day of therapy and a DLT occurred. Patients with premature termination of either azacitidine treatment and/or induction therapy during their first induction course were evaluable only if a DLT occurred in this patient, otherwise, such a patient would be replaced by another patient in this dose level. Patients received supportive care including standard antibiotic and antifungal prophylaxis according to each institution’s guidelines.

### Primary and Secondary Endpoints

The primary endpoint of this dose finding study was safety and toxicity of both dose levels. The recommended dose for the controlled part of the trial was defined as the highest dose level with one or less out of six evaluable patients experiencing a DLT. A separate dose level 0 with azacitidine 18 mg/sqm would have been evaluated in additional six patients in case of >1 DLT in each dose level. If >1 DLT would have been occurred also in this dose level 0, no azacitidine would have been administered preceeding the cytotoxic cycles in the controlled part of the trial. Adverse events (AE’s) and severe AE’s (SAE’s) were graded according to NCI common toxicity criteria (CTC) v3.0. CTCAE Grade 4 leukocytopenia, neutropenia, and thrombocytopenia as signs of active antileukemic treatment were assessed as an SAE only if persisting >42 days after the last application of cytotoxic chemotherapy in patients with blast clearance with (CR) or without (CRi) complete hematological recovery and then defined a DLT.

Assessment of treatment response was performed seven days after the end of the first induction cycle as described above, after hematological recovery following induction therapy, but not later than 35 days after the start of the last induction cycle, and every three months after achievement of complete remission until disease progression. Patients were monitored with physical examination, complete blood count and chemistry profile at least once weekly during induction and consolidation, and then at least monthly for the first two years after treatment start during follow-up. Response to treatment was defined according to the revised recommendations of the International Working Group for AML [14].

### Cytogenetic and Molecular Analysis

Cytogenetic analysis was performed by classical G-banding as previously described [15]. Molecular analysis for mutational status of Nucleophosmin (*NPM*), fms-like tyrosine kinase 3 (*FLT3*), additional sex combs like 1 (*ASXL1*), DNA methyltransferase 3A (*DNMT3A*), ten-eleven translocation 2 (*TET2*), isocitrate dehydrogenase 1 (*IDH1*) and 2 (*IDH2*) was performed as published [16], [17], [18], [19], [20], [21], [22]. Primers for *TET2* were modified from [23] and are listed in Table S1. Cytogenetic and molecular risk classification was performed according to the ELN 2010 criteria [24].

### Statistical Analysis

Overall survival was measured from the first day of azacitidine treatment until death of any course. Event-free survival was measured from the first day of treatment until therapy failure, relapse or death of any course. Patients without an event were censored at last follow-up. Follow-up time was calculated using reverse censoring. These outcome parameters were determined only exploratory for the total cohort. Statistical analyses were performed using IBM SPSS Statistics 20 for Windows (IBM Corporation, Somers, NY, USA).

## Results

### Patient Characteristics

**Figure 2:**
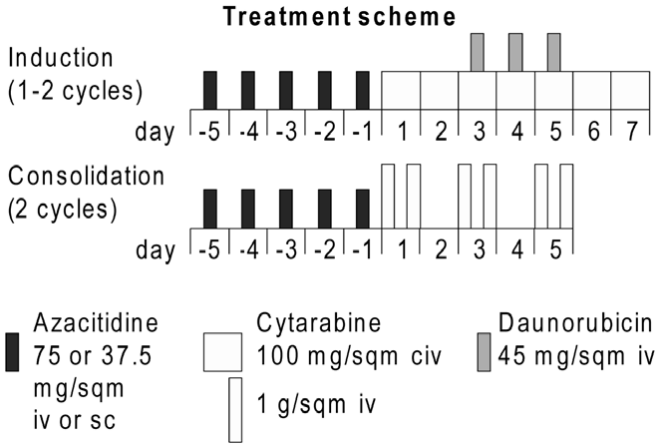
Outline of induction and consolidation therapy. Patients were randomised between 37.5 mg/sqm (dose level 1) and 75 mg/sqm (dose level 2) of azacitidine administered before each cycle of chemotherapy. Patients received 1–2 cycles of induction chemotherapy (‘7+3’) and responding patients received 2 cycles of consolidation therapy (intermediate-dose cytarabine).

Between September 2009 and February 2010, 12 patients were included in this pilot trial; four from two centers each, and three and one from the remaining centers, respectively. Data bank snapshot was taken on February 15th, 2012. The CONSORT statement of these patients is shown in Fig. 2. The clinical characteristics of these patients are depicted in Table 1. Median age was 68 years. Six patients were male. Median ECOG performance status was 1. According to ELN 2010 classification, two patients (no. 4 and 9) were low risk, four patients (no. 2, 7, 8 and 12) had intermediate-I risk, and three each intermediate-II (no. 3, 6 and 11) and high risk (no. 1, 5 and 10) (Table 1). Detected class III mutations are listed in Table 1 and Table S2.

**Table 1:** Patient characteristics and outcome of the 12 patients included into the trial.

PatientNo	age at diagnosis (years)	sex	AZA doselevel	type ofleukemia	PS	cytogenetics	Mutation status	Early BMassessment	Inductionresult	Survival time (days)	status
1	71	female	75	secondary	2	complex karyotype	b	n.a.	early death	8	
2	63	male	37.5	secondary	0	normal karyotype	*ASXL1*	Blast clearance	CR	616+	1st CR
3	67	male	75	de novo	1	trisomy 8		Blast clearance	CR	589+	1st CR
4	67	female	75	de novo	2	normal karyotype	*NPM1*	Blast persistance	CR	455	
5	67	male	37.5	secondary	1	monosomy 7		Blast clearance	early death	31	
6	70	female	75	secondary	1	trisomy 13, trisomy 22^a^		Blast persistance	CR	266	
7	76	female	37.5	de novo	2	normal karyotype		Blast clearance	early death	28	
8	74	male	75	secondary	1	normal karyotype	*TET2*	Blast persistance	refractory	246	
9	70	female	75	de novo	1	normal karyotype	*NPM1, ASXL1, IDH2*	Blast persistance	CR	215	
10	68	female	37.5	de novo	1	complex karyotype	b	Blast persistance	CR^c^	554	
11	68	male	37.5	de novo	0	trisomy 8	*ASXL1, IDH1, TET2*	Blast persistance	refractory	704+	CRi after relapse^d^
12	68	male	37.5	de novo	2	normal karyotype	*FLT3-ITD*	Blast persistance	CR	625+	CRi after relapse

Abbreviations: ASXL1, additional sex combs like 1; AZA, 5-azacitidine; BM, bone marrow; CR, complete remission; FLT3, fms-like tyrosine kinase 3; IDH, isocitrate dehydrogenase; ITD, internal tandem duplication; NPM1, Nucleophosmin 1; PS, ECOG performance status; TET2, ten-eleven translocation 2.

a Presence of a sole trisomy in each of two clones.

b Mutational analysis revealed wildtype status for NPM and no FLT3-ITD. Analysis of ASXL1, DNMT3A, IDH1, IDH2 and TET2 could not be performed due to lack of material.

c In patient 10, CR was achieved after trial termination without further therapy.

d Relapse after achievement of CR by salvage therapy.

### Safety and Toxicity

**Figure 3:**
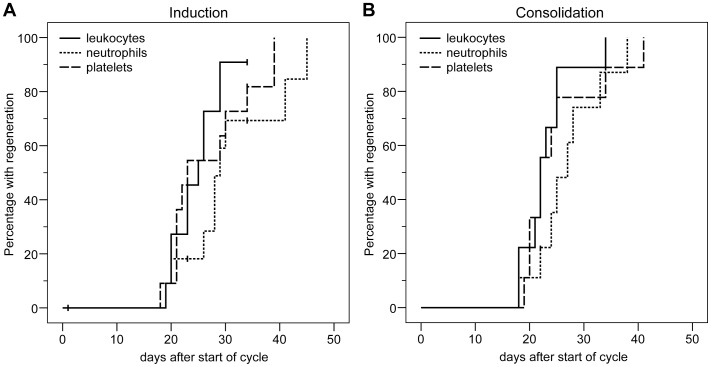
Hematotoxicity of induction and consolidation therapy. The time from start of chemotherapy until regeneration of leukocytes >1,000/µl, neutrophils >500/µl and plateletes >20,000/µl (transfusion independent) is shown. A: induction therapy, B: consolidation therapy.

No DLT occurred in either dose level. Nine severe adverse events occurred in five patients, two in the 75 mg/sqm dose level (two patients) and seven in the 37.5 mg/sqm dose level (three patients) (Table 2, labelled with ‘SAE: yes’). In two patients, the SAEs had a fatal outcome. 19 AE’s in ten patients were grade 3 or higher, seven in the 75 mg/sqm dose level and twelve of them in the 37.5 mg/sqm dose level (Table 2). With the exception of a neutropenic septicemia which occurred after the second consolidation course (patient 9), neutropenic fever in patient 3 and drug fever to cytarabine in both consolidation courses and diarrhea in one consolidation course of patient 4, all other SAEs and AEs grade 3 or 4 occurred during induction therapy. 162 AE’s of all grades occurred, among them 102 in the 75 mg/sqm dose level and 62 in the 37.5 mg/sqm dose level (Table S3). The most frequently occurring AE’s were: fever (11), peripheral edemas (8), diarrhea (8), nausea (8), vomiting (8), and exanthema (6). Infections (20 versus 7), AE’s of the cardiovascular (19 versus 7), musculoskelettal (5 versus 0) and gastrointestinal system (27 versus 22) were more frequent in the 75 mg/sqm compared to the 37.5 mg/sqm dose level. Among the AE’s of the cardiovascular system were peripheral edemas (5 versus 3), hypertension (2 versus 1), hypotension (2 versus 0), tachycardia (2 versus 1), chest pain (2 versus 0), bradycardia (1 versus 0), arrhythmia (1 versus 0), cardiac failure (Taku Tsuko cardiomyopathy, 0 versus 1), and vasovagal syncope (1 versus 0 of the 75 mg/sqm versus the 37.5 mg/sqm dose level, respectively) (Table S3). With the exception of one cardiac failure in the 37.5 mg/sqm arm (Table 2), all AE’s of the cardiovascular system were grade ≤2. Fig. 3 shows the time to regeneration after induction and after consolidation therapy. After induction therapy, median time to regeneration for patients in 37.5 mg/sqm and the 75 mg/sqm dose level was 23 and 25 days to a leukocyte count of >1,000/µl, 29 and 28 days to a neutrophil count >500/µl and 22 and 30 days to a transfusion-independent platelet count >20,000/µl, respectively (all comparisons not significant). In nine consolidation courses applied to five patients in CR, median time to regeneration was 22 days for leukocytes, 27 days for neutrophils and 22 days for platelets.

**Table 2:** Listing of all severe adverse events and adverse events grade 3 or 4.

Pat No	term	SAE	grade (CTCAE)	outcome	AZA dose level
1	bladder pain	no	3	resolved	75
1	pneumonia	**yes**	5	death	75
2	neutropenic fever	no	3	resolved	37.5
2	allergic exanthema	no	3	resolved	37.5
3	neutropenic fever	no	3	resolved	75
4	drug fever	no	3	resolved	75
4	diarrhea	no	3	resolved	75
4	drug fever	no	3	resolved	75
5	diarrhea	no	3	resolved	37.5
5	neutropenic fever	no	3	resolved	37.5
5	hyperbilirubinemia	**yes**	3	ongoing	37.5
5	Daunorubicin extravasation	**yes**	2	resolved	37.5
7	apoplectic stroke	**yes**	5	death^a^	37.5
7	Reduced general condition	no	4	ongoing	37.5
7	aseptic lymphocytic meningoencephalitis	**yes**	2	resolved	37.5
7	moist crackles	**yes**	5	death^a^	37.5
9	neutropenic septicemia	**yes**	4	resolved	75
10	acute hemolysis	**yes**	4	resolved	37.5
10	cardiac failure (Taku Tsuko cardiomyopathy)	**yes**	4	improved	37.5
11	neutropenic fever	no	3	resolved	37.5
12	neutropenic fever	no	3	resolved	37.5

The highest CTCAE grade of each event is listed. Hematotoxicity was not listed as adverse event unless persisting >42 days with grade IV after start of the last chemotherapy cycle without evidence of persisting leukemia.

Abbreviations: AZA, 5-azacitidine; CTCAE, common terminology criteria for adverse events; SAE, serious adverse event.

a In patient 7, both SAEs were documented with a fatal outcome.

### Drug Delivery

Seven patients received one and five patients received two induction courses. No patient had to be replaced due to hyperleukocytosis during the first azacitidine course as prespecified in the protocol. Reasons for applying only one induction course were complete blast clearance seven days after the end of the first induction cycle (four patients), early death due to pneumonia during the first course (one patient), progressive disease (one patient) and an SAE (one patient). Of all seven responding patients, four received both consolidation cycles. Two patients proceeded to allogeneic stem cell transplantation (one after induction, one after first consolidation) in first complete remission.

### Treatment Outcome

**Figure 4:**
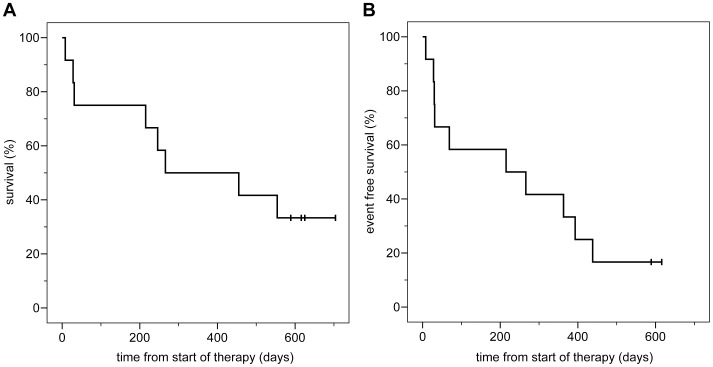
Outcome of the entire trial population. A: overall survival, B: event-free survival.

The treatment outcome is depicted in Table 1. In all patients, azacitidine was applied at the randomly allocated dose before the first cytotoxic induction treatment. One early death occurred during induction therapy before bone marrow evaluation. Of the remaining 11 patients, four showed an adequate blast clearance one week after the end of the induction cycle. Seven patients (four in dose level 75 mg/sqm, three in dose level 37.5 mg/sqm) achieved a CR after one (four patients) or two (three patients) induction courses whereas two additional patients succumbed to early death. One of the responding patients terminated study participation due to serious adverse events and achieved a CR without further therapy. Two patients had refractory disease after induction therapy, of whom one patient achieved a CR after termination of study participation and salvage therapy. After a median follow-up of 616 days from start of therapy, median overall and event-free survival of the entire cohort was 266 days and 215 days, respectively (Fig. 4).

Of note is the outcome of patient no. 10. The patient with a complex karyotype AML received one induction course with azacitidine 37.5 mg/sqm followed by ‘7+3’. One week after commencement of the first induction cycle, the bone marrow evaluation showed persisting infiltration with 77% blasts. Due to massive hemolysis (most likely fluorochinolone-induced) and subsequent cardiac failure, both regarded as a SAE, trial treatment was stopped on day 31 of therapy. Despite no further treatment after termination of study participation, the patient achieved a complete remission on day 69 (38 days after end of trial), while both SAE’s resolved. The patient remained in CR with good health and in the absence of further therapy for almost a year before relapse occurred.

Outcome by ELN risk group revealed 2 out of 2 patients achieving a CR with good risk, 2/4 patients with intermediate-I risk, 2/3 patients with intermediate-II risk and 1/3 patients with high risk.

## Discussion

We herein present data of a dose finding trial to evaluate safety, toxicity and feasibility of the addition of azacitidine to standard induction and consolidation therapy in older, medically fit patients with newly diagnosed AML. While intensive chemotherapy leads to a CR rate of 50% in this patient group, the probability of long-term survival is less than 10%, demonstrating the need for alternative and more efficacious treatment options. Azacitidine has a proven efficacy in patients with high-risk MDS and low-proliferating AML with 30% bone marrow blasts or less [8], [25], even though this treatment is still considered to be palliative. Therefore, it seems desirable to assess the efficacy of the drug in combination with standard chemotherapy. We decided to administer azacitidine before the cytotoxic therapy on the basis of existing preclinical data suggesting beneficial effects of azacitidine priming [10]. So far, azacitidine in a dosage of 75 mg/sqm has been evaluated in combination with high-dose cytarabine in pediatric patients with relapsed, cytarabine-refractory ALL [13].

Since a combination of azacitidine with a cytarabine and anthracyclines has not been evaluated, we assessed two different dose levels of azacitidine to determine the maximal tolerated dose.

The seven complete remissions seen in our pilot trial are in line with observed remission rates with intensive chemotherapy in this entity and age group, and the observed survival rates are encouraging.

Recent reports found an association with response to therapy in patients carrying DNMT3A, ASXL1 or TET2 mutations [26]–[28]. In our cohort, the frequency of those class III mutations was too low to draw any conclusions about a possible association with response.

Regarding toxicity, we observed three early deaths during induction therapy attributable to pneumonia, hepatorenal syndrome and apoplectic stroke, respectively. While the latter two toxicities are uncommon, the death rate of 25% after an intensive, cytarabine- and anthracycline-containing induction therapy in this patient population is in line with published results [29]. The majority of severe adverse events and adverse events grade 3 or higher occurred in the 37.5 mg/sqm dose level, suggesting that an increased azacitidine dose does not lead to an increased toxicity in the dose range tested.

In our pilot trial, both dose levels could be safely administered prior to standard cytotoxic induction and consolidation therapy. Therefore, the independent Data Monitoring Committee (DMC) suggested 75 mg/sqm for 5 days as the recommended dose for the randomised controlled phase II part of this trial (AML-AZA, clinicaltrials.gov identifier: NCT00915252). Eligible patients in this open-label, controlled part of the trial are randomised 1∶1 between the experimental or the control arm. Patients in the experimental arm receive azacitidine 5×75 mg/sqm prior to each induction and, if responding, consolidation therapy cycles as described above, and responding patients in the experimental arm additionally receive a maintenance therapy after consolidation with azacitidine 75 mg/sqm over 5 days s.c. q28 days for up to one year after initiation of therapy. Patients in the control arm directly receive induction therapy, consisting of 7+3 induction, and responding patients receive consolidation therapy as described above. No placebo-controlled design was chosen because of ethical considerations (treatment of patients with untreated AML with placebo for 5 days before initiating induction therapy). Sample size of this controlled phase II part was initially planned for 216 subjects (108 each arm), with event-free survival being the primary endpoint. However, recruitment is currently halted due to an increased rate of cardiac toxicity observed in the experimental arm after recruitment of 213 patients. The DMC recently stated that there is no concern regarding a further conduct of the randomised phase II trial, and results of this controlled part will be published separately. Taken together, our results of the pilot trial reported herein demonstrate that it might be feasible to add azacitidine 75 mg/sqm for 5 days to standard induction chemotherapy. This is in line with a recently published phase I trial combining standard induction therapy with the hypomethylating agent decitabine, which also showed feasibility and promising CR rates [30]. However, six patients treated in each dose level might not be sufficient to fully evaluate the safety profile of this combination, which is obvious considering the observed cardiac toxicity in the controlled part of the trial. The combination of novel treatment strategies with proven chemotherapy regimens warrants further study in older patients with AML.

## Supporting Information

Table S1 **Primer used for TET2 amplification and sequencing.**
(DOCX)

Table S2 **Mutational analysis for the genes ASXL1, DNMT3A, IDH1, IDH2 and TET2.** Abbreviations: ASXL1, additional sex combs like 1; bp, base pair; chr., chromosome; IDH, isocitrate dehydrogenase; TET2, ten-eleven translocation 2. No aberrations in exons 15-23 of the DNMT3A coding sequence were detected.
(DOCX)

Table S3 **List of all adverse events during study participation.** All adverse events occurring from signing the informed consent until end of study participation are listed. Neutropenia, leukopenia and thrombocytopenia were not considered as an adverse event unless persisting >42 days with grade 4 after the last chemotherapy cycle in patients responding to therapy with a CR or CRi.
(DOCX)

Checklist S1 **CONSORT checklist.**
(DOC)

Protocol S1 **Study protocol.**
(PDF)
