# Supplementary material for: Feasibility of Azacitidine Added to Standard Chemotherapy in Older Patients with Acute Myeloid Leukemia — A Randomised SAL Pilot Study
Source: PLoS One. 2012 Dec 31;7(12):e52695. doi: 10.1371/journal.pone.0052695 (PMC3534078; doi:10.1371/journal.pone.0052695)
Supplement: Table S1 — Primer used for TET2 amplification and sequencing. (DOCX) [file pone.0052695.s001.docx]

| **PCR pair** | **Exon** | **Sequence forward primer** | **Sequence reverse primer** |
| --- | --- | --- | --- |
| 1 | 4 | ATTCAACTAGAGGGCAGCCTTG | ACTGTGCGTTTTATTCCTCCAT |
| 2 | 4 | CAAGAAAGTAGAGGGTATTCC | GTAATTAGCACTTTTCCCCTC |
| 3 | 4 | CACATAACTGCAGTGGGCCTG | GTTAGAGGTCTGTGCGGAATTG |
| 4 | 4 | CCTGTGAGATCACTCACCCATC | TTTTGTTTTAAATACCGTTCAGAGC |
| 5 | 4 | AATTCTGTTCAGGTTCCAGCAG | ATGGATTAGGACTCTGGGAAGG |
| 6 | 4 | GGGAAGTGAAAATAGAGGGTAAAC | CCTGGTTTCAGATAGTGCTGTG |
| 7 | 4 | GATTCTGAAGGGTCGAGACAAG | CTGGAGATGTTGGTCCACTGTA |
| 8 | 4 | TCACAAATGTACCAAGTTGAAATG | GTTTGTGCTGCCTGTTTATGAG |
| 9 | 4 | TGAGCCATTTTCAAACTCACAC | GTACTTCCTCCAGTCCCATTTG |
| 10 | 4 | CAAAATCAAGCGAGTTCGAG | GTTGTGACTTCTGCTCCTGTTC |
| 11 | 4 | ATAATGTGATCCCAAAGCAAGA | TTTGGGGTTGCTGTGTTTG |
| 12 | 4 | AAAAGCATGCTGCTCTAAGGTG | AAGAGCCTTATGGTCAAATAACG |
| 13 | 4 | ATGGAGCAGCATCTGAAGCA | TGAGTCTTGACAGGTGTATCCAA |
| 14 | 4 | AACAGCTGCTTCTGTTCTCAAT | GACACAAGCATCGGTAACTTGA |
| 15 | 5 | TGCCTCTTGAATTCATTTGCTA | GTAACCCAATTCTCAGGGTCAG |
| 16 | 6 | GTGACCCTTGTTTTGTTTTGG | CAAAGATTGGGCTTTCCTATC |
| 17 | 7 | GCACAGCCTATATAATGCTATCCA | TGTCATATTGTTCACTTCATCTAAGC |
| 18 | 8 | AGGGGAATAATCTAACTGATAGTCTCT | TTTTTGGACATAGGTCATTAGTAACAA |
| 19 | 9 | GTTTTCGGTGTAAGAGTAAAACTAACT | AGTGTGAGAACAGACTCAACAGC |
| 20 | 10 | CCTGTAGTTGAGGCTGTAATGTC | CTAGTTTCCTTTGTCGGCAAGT |
| 21 | 10 | TGCCATTCAGGTACTGAGTTCT | AAGTTGATGGGGGCAAAAC |
| 22 | 11 | AAAGATACCTGTTTCTGTTCTCTCTT | CAGCTTGAGATGAGGTGGAATA |
| 23 | 11 | CACTTCAGATATCTATGGAAGCAC | TGCTGAAACCATCTCCCTGC |
| 24 | 11 | AGCCAAGGTTTGGAAATAGCC | GGGCATGAAGAGAGCTGTTG |
| 25 | 11 | TCTCACATAATCCATAACTACAGTGC | AATGTCAGGATCCAGAAAGCTC |
| 26 | 11 | AGAGGACAACGATGAGGTCTG | GTCTGGGCCATACTTTTCAC |
| 27 | 11 | AAGCCAAAATGGCTGAAAAAG | TTGTGGTCTTTTCAAGTGAGGT |

**Table S1. Primer used for TET2 amplification and sequencing.**
