# Supplementary material for: Feasibility of Azacitidine Added to Standard Chemotherapy in Older Patients with Acute Myeloid Leukemia — A Randomised SAL Pilot Study
Source: PLoS One. 2012 Dec 31;7(12):e52695. doi: 10.1371/journal.pone.0052695 (PMC3534078; doi:10.1371/journal.pone.0052695)
Supplement: Table S2 — Mutational analysis for the genes ASXL1, DNMT3A, IDH1, IDH2 and TET2. Abbreviations: ASXL1, additional sex combs like 1; bp, base pair; chr., chromosome; IDH, isocitrate dehydrogenase; TET2, ten-eleven translocation 2. No aberrations in exons 15-23 of the DNMT3A coding sequence were detected. (DOCX) [file pone.0052695.s002.docx]

| **Gene** | **Patient No** | **Aberration on base level** | **Nucleotide position** | **Effect on amino acid structure** |
| --- | --- | --- | --- | --- |
| ASXL1 | 2 | 23 bp deletion | starting at chr.20:31022415 | frameshift |
| ASXL1 | 9 | G**C**A>G**T**A | chr.20:31024450 | A1312V |
| ASXL1 | 11 | G**C**A>G**T**A | chr.20:31024450 | A1312V |
| IDH1 | 11 | **C**GT>**T**GT | chr.2:209113113 | R132C |
| IDH2 | 9 | C**G**G>C**A**G | chr.15:90631934 | R140Q |
| TET2 | 8 | 7 bp deletion | starting at chr.4:106157354 | frameshift |
| TET2 | 11 | **C**GA>**T**GA | chr.4:106164778 | R1216Stop |

**Table S2. Mutational analysis for the genes ASXL1, DNMT3A, IDH1, IDH2 and TET2.**Abbreviations: ASXL1, additional sex combs like 1; bp, base pair; chr., chromosome; IDH, isocitrate dehydrogenase; TET2, ten-eleven translocation 2
No aberrations in exons 15-23 of the DNMT3A coding sequence were detected
