# Supplementary material for: Feasibility of Azacitidine Added to Standard Chemotherapy in Older Patients with Acute Myeloid Leukemia — A Randomised SAL Pilot Study
Source: PLoS One. 2012 Dec 31;7(12):e52695. doi: 10.1371/journal.pone.0052695 (PMC3534078; doi:10.1371/journal.pone.0052695)
Supplement: Table S3 — List of all adverse events during study participation. All adverse events occurring from signing the informed consent until end of study participation are listed. Neutropenia, leukopenia and thrombocytopenia were not considered as an adverse event unless persisting >42 days with grade 4 after the last chemotherapy cycle in patients responding to therapy with a CR or CRi. (DOCX) [file pone.0052695.s003.docx]

| **AE Term** | DL1 (37.5mg/m2/day) | | | | | | | DL2 (75mg/m2/day) | | | | | | | **total both**  **cohorts** |
| --- | --- | --- | --- | --- | --- | --- | --- | --- | --- | --- | --- | --- | --- | --- | --- |
|  | patient no | | | | | | **sub-total** | patient no | | | | | | **sub-total** |  |
|  | 2 | 5 | 7 | 10 | 11 | 12 |  | 1 | 3 | 4 | 6 | 8 | 9 |  |  |
|  | N | N | N | N | N | N | **N** | N | N | N | N | N | N | **N** | **N** |
|  |  |  |  |  |  |  |  |  |  |  |  |  |  |  |  |
| **INFECTIONS** |  |  |  |  |  |  |  |  |  |  |  |  |  |  |  |
| fever of unknown origin | 1 | 1 | 1 |  | 1 | 1 | **5** | 1 | 1 | 1 | 1 | 1 | 1 | **6** | **11** |
| pneumonia |  |  |  | 1 |  |  | **1** | 1 |  |  |  | 1 | 1 | **2** | **3** |
| shivering |  |  |  |  |  |  |  |  |  |  | 1 | 1 | 1 | **3** | **3** |
| bacteriemia |  |  |  |  |  |  |  |  | 1 | 1 |  |  |  | **2** | **2** |
| follikulitis |  |  |  |  |  |  |  |  |  |  |  | 1 | 1 | **2** | **2** |
| soft tissue infection (CVC site) |  |  |  |  |  | 1 | **1** |  |  | 1 |  |  |  | **1** | **2** |
| vaginal Candida infection |  |  |  |  |  |  |  | 1 |  |  |  |  |  | **1** | **1** |
| erysipela |  |  |  |  |  |  |  |  | 1 |  |  |  |  | **1** | **1** |
| neutropenic septicemia |  |  |  |  |  |  |  |  |  |  |  |  | 1 | **1** | **1** |
| perichondritis |  |  |  |  |  |  |  |  |  |  | 1 |  |  | **1** | **1** |
|  |  |  |  |  |  |  |  |  |  |  |  |  |  |  |  |
| **BLEEDINGS** |  |  |  |  |  |  |  |  |  |  |  |  |  |  |  |
| epistaxis |  | 1 |  |  |  |  | **1** |  | 1 | 1 | 1 |  |  | **3** | **4** |
| petechia |  | 1 |  |  |  | 1 | **2** |  |  |  | 1 |  | 1 | **2** | **4** |
| hematuria |  |  | 1 |  |  |  | **1** |  |  |  |  |  |  |  | **1** |
| hematoma |  |  |  |  |  |  |  |  |  |  |  | 1 |  | **1** | **1** |
| melena |  |  |  |  |  |  |  |  | 1 |  |  |  |  | **1** | **1** |
|  |  |  |  |  |  |  |  |  |  |  |  |  |  |  |  |
| **EYES** |  |  |  |  |  |  |  |  |  |  |  |  |  |  |  |
| conjunctivitis |  |  |  |  |  | 1 | **1** |  |  |  |  |  |  |  | **1** |
| dry eyes |  |  |  |  |  |  |  |  |  |  | 1 |  |  | **1** | **1** |
| painful right eye |  |  |  |  |  |  |  |  |  |  |  |  | 1 | **1** | **1** |
|  |  |  |  |  |  |  |  |  |  |  |  |  |  |  |  |
| **BLOOD SYSTEM** |  |  |  |  |  |  |  |  |  |  |  |  |  |  |  |
| acute hemolysis |  |  |  | 1 |  |  | **1** |  |  |  |  |  |  |  | **1** |
|  |  |  |  |  |  |  |  |  |  |  |  |  |  |  |  |
| **SKIN** |  |  |  |  |  |  |  |  |  |  |  |  |  |  |  |
| exanthema | 1 |  | 1 |  |  |  | **2** |  |  | 1 | 1 | 1 | 1 | **4** | **6** |
| pruritus |  |  | 1 |  | 1 |  | **2** |  |  |  | 1 |  |  | **1** | **3** |
| erythema at azacitidin injection site |  |  |  |  |  |  |  |  | 1 |  |  |  | 1 | **2** | **2** |
| allergic reaction to thrombocyte transfusion |  |  |  | 1 |  |  | **1** |  |  |  |  |  |  |  | **1** |
| skin defect left lower leg |  |  |  |  |  |  |  |  | 1 |  |  |  |  | **1** | **1** |
| daunorubicin extravasation |  | 1 |  |  |  |  | **1** |  |  |  |  |  |  |  | **1** |
| allergic vasculitis |  |  |  |  |  |  |  |  |  |  | 1 |  |  | **1** | **1** |
| dry skin, rhagades |  |  | 1 |  |  |  | **1** |  |  |  |  |  |  |  | **1** |
| angioedema |  |  |  |  |  |  |  |  |  |  |  |  | 1 | **1** | **1** |
|  |  |  |  |  |  |  |  |  |  |  |  |  |  |  |  |
| **CARDIOVASCULAR SYSTEM** |  |  |  |  |  |  |  |  |  |  |  |  |  |  |  |
| peripheral edema |  | 1 | 1 |  |  | 1 | **3** | 1 |  | 1 | 1 | 1 | 1 | **5** | **8** |
| hypertension |  |  | 1 |  |  |  | **1** |  | 1 | 1 |  |  |  | **2** | **3** |
| tachycardia |  |  | 1 |  | 1 |  | **2** |  |  |  |  |  | 1 | **1** | **3** |
| hypotension |  |  |  |  |  |  |  |  |  |  | 1 |  | 1 | **2** | **2** |
| thrombophlebitis |  |  |  |  |  |  |  |  |  |  | 1 |  | 1 | **2** | **2** |
| chest pain |  |  |  |  |  |  |  |  |  |  | 1 |  | 1 | **2** | **2** |
| bradycardia |  |  |  |  |  |  |  |  |  |  |  |  | 1 | **1** | **1** |
| cardiac failure (Taku Tsuko cardiomyopathy) |  |  |  | 1 |  |  | **1** |  |  |  |  |  |  |  | **1** |
| deep vein thrombosis |  |  |  |  |  |  |  | 1 |  |  |  |  |  | **1** | **1** |
| perianal venous thrombosis |  |  |  |  |  |  |  |  |  | 1 |  |  |  | **1** | **1** |
| arrhythmia |  |  |  |  |  |  |  |  |  |  |  |  | 1 | **1** | **1** |
| vasovagal syncope |  |  |  |  |  |  |  |  | 1 |  |  |  |  | **1** | **1** |
|  |  |  |  |  |  |  |  |  |  |  |  |  |  |  |  |
| **GI TRACT** |  |  |  |  |  |  |  |  |  |  |  |  |  |  |  |
| diarrhea |  | 1 | 1 |  | 1 |  | **3** |  | 1 | 1 | 1 | 1 | 1 | **5** | **8** |
| vomiting |  | 1 | 1 | 1 |  |  | **3** | 1 | 1 | 1 | 1 |  | 1 | **5** | **8** |
| nausea |  | 1 | 1 | 1 | 1 | 1 | **5** |  | 1 | 1 |  |  | 1 | **3** | **8** |
| mucositis | 1 | 1 |  |  | 1 |  | **3** |  |  | 1 |  | 1 |  | **2** | **5** |
| anorexia |  |  | 1 | 1 |  |  | **2** |  |  |  |  |  | 1 | **1** | **3** |
| obstipation |  |  |  |  |  |  |  |  |  |  | 1 | 1 | 1 | **3** | **3** |
| hemorrhoids |  |  |  |  |  |  |  |  |  |  | 1 |  | 1 | **2** | **2** |
| heartburn |  |  |  | 1 |  |  | **1** |  |  |  |  |  | 1 | **1** | **2** |
| hyperbilirubinemia |  | 1 |  |  |  |  | **1** |  |  |  |  |  |  |  | **1** |
| liver enzyme elevation |  |  |  |  |  |  |  |  | 1 |  |  |  |  | **1** | **1** |
| abdominal pain |  |  |  |  |  |  |  |  |  |  | 1 |  |  | **1** | **1** |
| xerostomia |  |  |  |  |  |  |  |  |  |  |  |  | 1 | **1** | **1** |
| swallowing difficulties | 1 |  |  |  |  |  | **1** |  |  |  |  |  |  |  | **1** |
| faecal incontinence |  |  | 1 |  |  |  | **1** |  |  |  |  |  |  |  | **1** |
| feeling of fullness |  |  | 1 |  |  |  | **1** |  |  |  |  |  |  |  | **1** |
| coated tongue |  |  |  |  |  |  |  |  |  | 1 |  |  |  | **1** | **1** |
| tongue ulcer |  |  |  |  |  | 1 | **1** |  |  |  |  |  |  |  | **1** |
| flatulence |  |  |  |  |  |  |  |  |  |  | 1 |  |  | **1** | **1** |
|  |  |  |  |  |  |  |  |  |  |  |  |  |  |  |  |
| **RESPIRATORY TRACT** |  |  |  |  |  |  |  |  |  |  |  |  |  |  |  |
| dyspnea |  |  | 1 |  |  | 1 | **2** |  | 1 |  |  |  | 1 | **2** | **4** |
| coughing |  |  | 1 |  |  |  | **1** |  |  | 1 |  |  |  | **1** | **2** |
| pleuritic pain |  |  |  | 1 |  |  | **1** |  |  |  |  |  |  |  | **1** |
| coryza |  |  |  |  |  |  |  |  |  |  |  |  | 1 | **1** | **1** |
| moist crackles |  |  | 1 |  |  |  | **1** |  |  |  |  |  |  |  | **1** |
| dry nasal mucosa |  |  |  |  |  |  |  |  |  |  | 1 |  |  | **1** | **1** |
|  |  |  |  |  |  |  |  |  |  |  |  |  |  |  |  |
| **MUSCULOSKELETTAL SYSTEM** |  |  |  |  |  |  |  |  |  |  |  |  |  |  |  |
| back pain |  |  |  |  |  |  |  |  |  | 1 |  | 1 |  | **2** | **2** |
| painful left zygomatic bone |  |  |  |  |  |  |  |  |  |  | 1 |  |  | **1** | **1** |
| painful left leg after bone marrow puncture |  |  |  |  |  |  |  |  |  |  | 1 |  |  | **1** | **1** |
| bilateral inguinal pain |  |  |  |  |  |  |  |  |  |  |  |  | 1 | **1** | **1** |
|  |  |  |  |  |  |  |  |  |  |  |  |  |  |  |  |
| **NEUROLOGY / PSYCHIATRY** |  |  |  |  |  |  |  |  |  |  |  |  |  |  |  |
| cephalgias |  |  |  |  |  | 1 | **1** |  |  | 1 | 1 |  |  | **2** | **3** |
| fatigue |  |  |  | 1 |  |  | **1** |  |  |  |  |  | 1 | **1** | **2** |
| unconsciousness |  |  | 1 |  |  |  | **1** |  |  |  |  |  |  |  | **1** |
| disorientation |  |  |  |  |  |  |  |  | 1 |  |  |  |  | **1** | **1** |
| apoplectic stroke |  |  | 1 |  |  |  | **1** |  |  |  |  |  |  |  | **1** |
| insomnia |  |  |  |  |  | 1 | **1** |  |  |  |  |  |  |  | **1** |
| dizziness |  |  |  |  |  |  |  |  |  |  |  |  | 1 | **1** | **1** |
| aseptic lymphocytic meningoencephalitis |  |  | 1 |  |  |  | **1** |  |  |  |  |  |  |  | **1** |
| polyneuropathy |  |  |  |  |  |  |  |  |  |  |  |  | 1 | **1** | **1** |
|  |  |  |  |  |  |  |  |  |  |  |  |  |  |  |  |
| **UROGENITAL TRACT** |  |  |  |  |  |  |  |  |  |  |  |  |  |  |  |
| bladder pain |  |  |  |  |  |  |  | 1 |  |  |  |  |  | **1** | **1** |
| urine incontinence |  |  | 1 |  |  |  | **1** |  |  |  |  |  |  |  | **1** |
|  |  |  |  |  |  |  |  |  |  |  |  |  |  |  |  |
| **GENERAL** |  |  |  |  |  |  |  |  |  |  |  |  |  |  |  |
| reduced general appearance |  |  | 1 |  |  |  | **1** |  |  |  |  |  |  |  | **1** |
|  |  |  |  |  |  |  |  |  |  |  |  |  |  |  |  |
|  |  |  |  |  |  |  |  |  |  |  |  |  |  |  |  |
| **TOTAL** | 4 | 10 | 22 | 10 | 6 | 10 | **62** | 7 | 15 | 16 | 23 | 11 | 31 | **102** | **164** |
|  |  |  |  |  |  |  |  |  |  |  |  |  |  |  |  |

**Table S3. List of all adverse events during study participation.**

**All adverse events occurring from signing the informed consent until end of study participation are listed. Neutropenia, leukopenia and thrombocytopenia were not considered as an adverse event unless persisting >42 days with grade 4 after the last chemotherapy cycle in patients responding to therapy with a CR or CRi.**
