# Supplementary material for: Feasibility of Azacitidine Added to Standard Chemotherapy in Older Patients with Acute Myeloid Leukemia — A Randomised SAL Pilot Study
Source: PLoS One. 2012 Dec 31;7(12):e52695. doi: 10.1371/journal.pone.0052695 (PMC3534078; doi:10.1371/journal.pone.0052695)
Supplement: Protocol S1 — Study protocol. (PDF) [file pone.0052695.s005.pdf]

A randomized, multi-center phase II trial to assess the efficacy of 5-azacytidine added to standard primary therapy in elderly patients with newly diagnosed AML

A randomized, multi-center phase II study with a preceding dose finding

Study protocol

November 2008

Version 3.0

CONFIDENTIAL

**Sponsor:**	Universitätsklinikum Münster (UKM)
**Co-ordinating Investigator:**	Prof. Dr. med. C. Müller-Tidow
**EudraCT Number:**	2008-004583-40
**Protocol Code:**	101010
**Running Title:**	AML-AZA

Sponsor: **Universitätsklinikum Münster**
Dr. rer. pol. C. Hoppenheit
Domagkstr. 5
48149 Münster

____
(Date and Signature)

Co-ordinating Investigator: **Prof. Dr. med. C. Müller-Tidow**
Universitätsklinikum Münster
Klinik und Poliklinik A
Albert-Schweitzer-Str. 33
48149 Münster

____
(Date and Signature)

Statistician: **Dr. rer. nat. J. Gerß**
Institut für Medizinische Informatik und Biomathematik
der Universität und des Universitätsklinikums Münster
Domagkstr. 9
48149 Münster

____
(Date and Signature)

TABLE OF CONTENTS

1 ABBREVIATIONS 7

2 BACKGROUND INFORMATION / INTRODUCTION 9

2.1 Condition background and current treatment 9

2.2 Molecular pathogenesis 9

2.3 Investigational product background 10

2.3.1 Preclinical information 10

2.3.2 Clinical information 11

3 STUDY OBJECTIVES 14

3.1 Rationale for this study 14

3.1.1 Benefit / risk ratio 14

3.2 Study objectives 15

3.2.1 Primary 15

3.2.2 Secondary 15

3.3 Study design 16

4 SELECTION, RANDOMIZATION AND WITHDRAWAL OF PATIENTS 16

4.1 Number of patients 16

4.2 Admission criteria 16

4.2.1 Inclusion criteria 16

4.2.2 Exclusion criteria 16

4.3 Registration, randomization and stratification 17

4.3.1 Registration 17

4.3.2 Stratification and Randomization Procedure 18

4.4 Withdrawal of patients 18

5 STUDY, STANDARD AND CONCOMITANT TREATMENTS 19

5.1 Investigational product 19

5.1.1 Relevant physical, chemical and pharmaceutical properties 19

5.1.2 Instructions for storage and handling 19

5.1.3 Interactions with concomitant medications 20

5.1.4 Drug accountability 20

5.2 Standard cytotoxic therapy 20
5.2.1 Cytarabine (AraC) 20
5.2.2 Daunorubicin 21
5.3 Treatment 22
5.3.1 Dose finding for 5-azacytidine during the combination therapy 22
5.3.2 Controlled phase - Induction therapy 24
5.3.3 Consolidation therapy 25
5.3.4 Maintenance therapy 26
5.3.5 Dose modification and delays of 5-azacytidine 27
5.4 Concomitant medications / therapy 29
6 STUDY PROCEDURES 30
6.1 Study evaluations 30
6.1.1 Evaluations before treatment 30
6.1.2 Evaluations during treatment 31
6.1.3 Evaluations at the end of maintenance therapy 31
6.1.4 Evaluations in case of relapse 32
6.1.5 Evaluations during follow up (standard routine parameters) 32
6.2 Schedule of evaluations 32
6.3 Bone marrow diagnostics including central diagnostics 35
6.3.1 Cytomorphologic examination and Cytochemistry 35
6.3.2 Immunphenotyping 36
6.3.3 Cytogenetics 36
6.3.4 Molecular genetic analyses 37
6.3.5 Central bone marrow diagnostics and shipment modalities 37
6.3.6 Methylation analysis 38
6.4 Duration of the study 39
6.5 End of study 39
6.6 Criteria for removal from study / premature end of study 39
6.6.1 Individual reasons (Criteria for removal of patients) 39
6.6.2 General reasons 39
7 ADVERSE EXPERIENCES 40
7.1 Definitions 40
7.1.1 Adverse Event (AE) 40

7.1.2 Adverse Reaction (AR) 40
7.1.3 Unexpected Adverse Reaction (UAR)..40
7.1.4 Definition of Serious Adverse Event (SAE) or Serious Adverse Reaction (SAR).........41
7.1.5 Suspected Unexpected Serious Adverse Reaction (SUSAR).......................................42

7.2 Period of observation, documentation and reporting...43

7.3 Warnings and Precautions 45
7.3.1 Investigational product (5-azacytidine) 45
7.3.2 Standard cytotoxic therapy (Cytarabine and Daunorubicin)......................................49

8 STUDY OUTCOME AND STATISTICAL ANALYSIS 50

8.1 Power and Sample Size Calculation 50

8.2 Statistical Analysis Plan...50

8.3 Number of patients..51

8.4 Efficacy...51
8.4.1 Response criteria...51

8.5 Definition of Study Endpoints...53

9 QUALITY ASSURANCE...54

9.1 Safety Committee 54

9.2 Reference Laboratories 54
9.2.1 Reference diagnostics for cytomorphology 54

9.3 Monitoring...55

9.4 Data Management 55

10 INVESTIGATOR'S RESPONSIBILITIES, ETHICAL CONSIDERATIONS, ...
CONFIDENTIALITY, INSURANCE..56

10.1 Investigator's responsibilities..56
10.1.1 Declaration of Helsinki and GCP compliance...56
10.1.2 Protocol adherence 56
10.1.3 Documentation and retention of records..56
10.1.4 Competent local authorities 56

10.2 Ethical considerations...57
10.2.1 Institutional Review Board / Independent Ethics Committee approval 57
10.2.2 Informed Consent...57

10.3 Confidentiality 57

10.4 Insurance 58

11 REFERENCES 59

12 APPENDICES 61

12.1 Synopsis of the study (in German) 61

12.2 Performance Status 64

12.3 Therapy protocols overview 65

1 ABBREVIATIONS

AE	Adverse event
ALT	Alanine transaminase
AML	Acute myeloid leukemia
AMLCG	AML Cooperative Group
AP	Alkaline phosphatase
APL	Acute promyelocyticleukaemia
AR	Adverse reaction
AraC	Cytosine arabinoside
AST	Aspartate transaminase
ATU	Temporary authorization for use
ATIII	Antithrombin III
CALGB	Cancer and Leukemia Group B
CBC	Complete blood count
CNS	Central nervous system
CpG-islands	Cytosine-phospho-guanine islands
CR	Complete remission
CRc	Complete cytogenetic remission
CRm	Complete moleculargenetic remission
CRF	Case report form
CT	Chemotherapy
CTCAE	Common terminology criteria for adverse events
CYP	Cytochrom P
DL	Dose level
DNA	Desoxyribonucleic acid
Dnmt	DNA methyltransferase
ECG	Electrocardiogram
ECOG	Eastern Cooperative Oncology Group
EFS	Event free survival
FAB	French-American-British cooperative group
Flt	Fms like tyrosine kinase
FISH	Fluorescence in situ hybridization
GCP	Good clinical practice
G-CSF	Granulocyte colony stimulating factor
GI	Gastrointestinal
HiDAC	High dose-cytarabine
HIV	Human immunodeficiency virus
IB	Investigator`s brochure
ICH	International Conference on Harmonization
IRB/IEC	Institutional Review Board/ Independent Ethics Committee

ITD	Internal tandem duplication
LDH	Lactatedehydrogenase
MDS	Myelodysplastic syndrome
MI	Myocardial infarction
MRD	Minimal residual disease
MUGA	Multiple Gated Acquisition Scan
NCI	National Cancer Institute
NPM	Nucleophosmin
NYHA	New York Heart Association
OS	Overall survival
PAT	Patienten
PB	Peripheral blood
PTT	Partial thromboplastin time
PVC	Polyvinyl chloride
RFS	Relapse free survival
RNA	Ribonucleic acid
SAE	Serious adverse event
SC	Subcutaneous injection
SUSAR	Suspected unexpected serious adverse reaction
t_{max}	Time to maximal plasma concentration
UAR	Unexpected adverse reaction
ULN	Upper limit of normal
WHO	World Health Organization
WWU	Westfälische Wilhelms Universität
ZKS	Zentrum für Klinische Studien Münster

2 BACKGROUND INFORMATION / INTRODUCTION

2.1 Condition background and current treatment

Acute Myeloid Leukemia (AML) is a clonal, malignant disorder that results from genetic and epigenetic changes in pluripotent stem or slightly more differentiated progenitor cells. The aberrant cells gain a growth and/or survival advantage in relationship to the normal pool of stem cells. Fewer than 30% of adults with AML can be expected to survive three or more years and be cured (1). Adverse prognostic factors include age over 60 years, poor ECOG performance status prior to therapy, secondary AML, a white cell count of more than 20,000/µl or an elevated serum lactate dehydrogenase at presentation. Detailed cytogenetic analysis of the leukemic blasts has also been demonstrated to provide critical prognostic information (2). Initial AML treatment is divided into two phases: induction and consolidation. With the use of cytarabine and an anthracycline as induction therapy, complete remissions can be routinely induced in 70%-80% of patients 60 years and younger and in approximately 50% of older patients (2). Consolidation therapy following the induction of an initial complete response is essential to prevent relapse. Three options are available: allogeneic or autologous bone marrow transplantation or chemotherapy. Transplantation is more often used in young patients with adverse karyotypes and chemotherapy in good prognosis patients (2). Non-transplant consolidation chemotherapy regimens commonly contain cytarabine. There appears to be a clear benefit in survival to patients younger than 60 years of age that received high-dose cytarabine regimens as consolidation (3).

2.2 Molecular pathogenesis

In the last decades, significant progress has been made about the molecular events inducing leukemic transformation in AML (reviewed in 4). Although AML is a heterogeneous disease, common features of the leukemic blasts include proliferative potential, increased stem cell self renewal and a block in differentiation at a relatively immature state, within the mitotic pool. It is now widely accepted that AML blasts already harbor several transforming events when the disease becomes clinically apparent. Many AML-specific oncogenic mutations and epigenetic alterations have been identified and their ability to cause leukemia has been analyzed in primary and human xenograft mouse models. A model has been suggested that at least two mutations from different complementation classes have to accumulate in a myeloid progenitor cell to cause AML (5). One of these mutations is thought to cause deregulation of transcriptional programs needed for the orchestration of myeloid differentiation. Thus, more than 50% of all AML cases have been shown to contain a mutation in a transcriptional regulator, often as a result from balanced reciprocal translocations.

These mutations often coincide with mutations in signal transduction mediators. Again, about 50% of AML cases have been shown to contain a mutation in a signaling mediator, most frequently in receptor tyrosine kinases or in the Ras oncogene. The receptor tyrosine kinase Flt3 has been found to be the most common target among these, in 30% of all AML cases. Flt3 mutations have been shown in several *in vitro* and animal models to cause malignant transformation of myeloid cells and to cooperate with other known oncogenes to cause an AML-type disease in mice.

In addition, it has been recently discovered that AML blasts show a profound disruption of epigenetic events, i.e. of the CpG island methylation status of promoters of a growing number of genes leading to an aberrant silencing of these genes (reviewed in 7). Importantly, epigenetic alterations occur far more frequently in AML blasts then genetic mutations. Treatment with demethylating agents *in vitro* leads to a reversal of the aberrant DNA methylation and to a partial differentiation of leukemic blasts (7).

In summary, AML is thought to be the result of multiple genetic and epigenetic events mediating a block of differentiation, inhibition of apoptosis and enhanced stem cell self renewal.

2.3 Investigational product background

5-azacytidine is a ring analog of the pyrimidine nucleoside cytidine. In dosages approved for the treatment of myelodysplastic syndromes it inhibits the methylating enzyme DNA methyltransferase leading to a demethylation of gene promoters and the reversal of aberrant gene silencing. For full details of the pre-clinical and clinical information please refer to the summary of product characteristics and the investigators brochure.

2.3.1 Preclinical information

2.3.1.1 General information

5-azacytidine is a ring analog of the naturally occurring pyrimidine nucleoside, cytidine. The synthesis of 5-azacytidine was first reported in 1964 by Piskala and Sorm at the Institute of Organic Chemistry and Biochemistry in Prague, Czechoslovakia. Two years later, scientists at the Upjohn Company (now Pfizer) reported the isolation of 5-azacytidine from Streptoverticilium ladakanus.

High concentrations of 5-azacytidine lead to an incorporation of the substance into DNA and a subsequent inhibition of DNA synthesis in the S-phase of the cell cycle, mimicking the mechanism of action of other pyrimidine-analogues such as cytarabine (reviewed in 4). In low concentrations, 5-azacytidine blocks cytosine methylation by noncompetitive inhibition of DNA methyltransferase (8).

2.3.1.2 Preclinical pharmacokinetics

Preclinical pharmacokinetics with 5-azacytidine have been carried out in the 1970's and 1980's in mice, rats and dogs according to the existing guidelines and standards in place during that time. Since

then, 5-azacytidine has been widely used in clinical studies in patients with myelodysplastic syndromes and acute myeloid leukemias and this preclinical pharmacokinetic data are superseded by now available clinical pharmacokinetic data which are listed below.

2.3.1.3 Preclinical toxicology

The preclinical toxicity profile of 5-azacytidine can be summarized as:

- Toxicology studies in mice, rats, dogs, and Rhesus monkeys identified the bone marrow, liver, kidneys, and lymphoid tissues (spleen, lymph nodes) as the main target organs of toxicity.
- The lethal dose of a single intravenous 5-azacytidine administration in mice, rats, and dogs was approximately 250 mg/m^2. Repeated daily dosing appears to increase the toxicity of 5-azacytidine.
- The potential carcinogenicity of 5-azacytidine was investigated in mice and rats. 5-azacytidine induced tumors of the hematopoietic system in female rats after intraperitoneal injection three times weekly over one year. In mice, an increased incidence of tumors of the lymphoreticular system, lung, mammarial gland and skin was observed. In male rats an increased incidence of testicular cancer was observed compared to the control group.
- The genotoxicity of 5-azacytidine is consistent with that of other nucleoside analogs that interact with nucleic acids. Likewise, similar to other agents with cytostatic properties, 5-azacytidine was embryotoxic and reduced the reproductive performance in mice and rats. It is important to note that animal study data is superseded in many respects by the extensive clinical safety data collected in the last two decades.

2.3.2 Clinical information

During the 1970s and 1980s, 5-azacytidine was investigated in Europe and the US as a treatment for a variety of malignancies, both as a single agent and in combination therapy. 5-azacytidine has been administered by intravenous (continuous infusion or bolus injection) or subcutaneous routes. Because of its clinical activity in relapsed refractory AML (referred to acute non-lymphocytic leukemia [ANLL] at the time), 5-azacytidine has been available from the National Cancer Institute (NCI) for single-agent induction therapy for adults and children with relapsed/refractory AML under the Group C Guidelines since 1975.

In 1985, the Cancer and Leukemia Group B (CALGB) study investigators began clinical trials with azacytidine in MDS patients under the auspices of the (NCI). Results from the three studies conducted by the CALGB (Protocols 8421, 8921, and 9221) have been published. The first two CALGB studies (Protocol 8421 and Protocol 8921) were uncontrolled phase II investigations. The most recent CALGB study (Protocol 9221) was a phase III investigation that compared 5-azacytidine to supportive care alone. The 5-azacytidine dose investigated in the CALGB studies was 75 mg/m^2/day for 7 days, repeated on a 28-day cycle. 5-azacytidine was administered by continuous IV infusion in the first study (Protocol 8421), and by SC injection in the two studies that followed. The dose was adjusted

based on toxicity and clinical response. In the phase III investigation (Protocol 9221), 5-azacytidine produced higher response rates than supportive care alone. In addition, 5-azacytidine prolonged the time to transformation to AML or death.

The efficacy of 5-azacytidine to treat MDS was also evaluated in 7 open-label studies conducted outside the CALGB protocols. The dosage regimen used in 6 of these studies was 75 mg/m^2 given daily for 7 days every 3-4 weeks by SC injection in 4 studies, SC or IV in 1 study, and the route was not specified in the last study. The dosage regimen used in the seventh study was 5-35 mg/m^2/day given by continuous IV infusion for 14 days. The lowest response rate was found in this seventh study, which suggests the mechanism of 5-azacytidine's activity requires repeated administration of a minimally effective dose to achieve improvement in hematological parameters.

Similar to other antimetabolites, bone marrow suppression (leukopenia, thrombocytopenia) is a common adverse event associated with 5-azacytidine. However, myelosuppression generally occurs more often and with greater severity at doses higher than those currently used to treat MDS. Gastrointestinal toxicity (i.e. nausea, vomiting, and diarrhea) can limit the dose of 5-azacytidine in any patient population. Infrequent adverse effects include neuromuscular aches, generalized weakness, renal tubular acidosis, and liver enzyme abnormalities. Erythema and burning at the injection site can occur following SC administration, which usually resolves within 24-72 hours.

Within the French ATU program, 108 patients with AML have been treated to date with 5-azacytidine monotherapy (9). Treatment consisted of 5-azacytidine 75 mg/m^2 s.c. for 5 days every 4 weeks. An update of 93 evaluable patients was presented at the 2007 meeting of the American Society of Hematology: Overall, 13% complete remissions and 36% partial remissions were observed with an overall response rate of 49%.

2.3.2.1 Combination with chemotherapeutic agents

5-azacytidine was combined with high-dose cytarabine in a phase I study of 17 pediatric patients with relapsed ALL after previous high-dose cytarabine treatment (10). In this study, 5-azacytidine was given in doses of 150 mg/m^2 as a continuous infusion over 5 days before treatment with high-dose cytarabine. No relevant excess toxicity was observed, and pretreatment with 5-azacytidine before high-dose cytarabine was able to revert cytarabine resistance and induced complete remission in 2 patients.

In a currently recruiting phase I/II combination study with 5-azacytidine and cytarabine in patients with relapsed AML, patients are randomized to 2 dose levels of 5-azacytidine followed by 2 dose levels of cytarabine (11). Patients are treated with either 37.5 or 75 mg/m^2 5-azacytidine intravenous for 7 days, followed by cytarabine either 100 mg/m^2 continuous intravenous infusion for 7 days or 1g/m^2 continuous intravenous infusion over 4 days, followed by a maintenance therapy with 5-azacytidine upon achievement of a complete remission. Twenty-seven patients with AML have been

enrolled so far, with one dose limiting toxicity (grade 3 mucositis) in a patient treated with 75mg/m^2 5-azacytidine and 100mg/m^2 cytarabine. No other dose limiting toxicities were observed so far.

2.3.2.2 Clinical pharmacokinetics

- Fast absorption after subcutaneous injection with a t_{max} of 0.5 hours
- High bioavailability of 88.6 % (70.2 % – 111.9 %) after subcutaneous injection
- Medium elimination of 5-azacytidine from plasma with a half-life of 0.69 ± 0.14 hours after subcutaneous injection
- Intracellular hydrolyzation to the inactive metabolites D-ribose and 5-azacytosine. Further metabolites are: 5-azauracil, ribosylguanylurea and ribosylazalidon, all inactive.
- The distribution volume is 76 l after intravenous injection; uptake can be detected into leukocytes and erythrocytes. Penetration to the blood-brain barrier and binding to plasma proteins has not sufficiently been investigated to date
- In vitro data suggest no inhibition or induction of CYP isoforms by 5-azacytidine. Studies about drug-drug interactions have not been performed
- Excretion of the compound is primarily via glomerular filtration into the urine (app. 90 %) with a clearance of 167 ± 49 hours

3 STUDY OBJECTIVES

3.1 Rationale for this study

Despite recent advances into the insight of the molecular mechanism of acute myelogenous leukemia, not much improvement in the still unsatisfactory treatment of patients > 60 years has been made with standard therapy of cytarabine and antracyclines, followed by intermediate-dose cytarabine consolidation.

Methylation of CpG islands within the promoter of genes by DNA methyltransferases (Dnmt) inhibits the expression of target genes. Gene silencing by aberrant promoter hypermethylation is a very frequent event in AML (7). 5-azacytidine is a compound with hypomethylating activity through Dnmt inhibition. *In vitro*, 5-azacytidine restores aberrant gene silencing mediated by promoter hypermethylation.

A poor outcome of acute myelogenous leukemia is often mediated by resistance against the antimetabolite cytosine-arabinoside. *In vitro* data have shown that resistance against cytosine arabinoside can be mediated by inactivation of deoxycytidine kinase (12). Preincubation of leukemic cells resistant to cytosine arabinoside with 5-azacytidine restores the activity of deoxycytidine kinase and renders these cells sensitive towards cytosine-arabinoside (12). As presented above, 5-azacytidine shows substantial clinical activity as monotherapy in high-risk AML patients and shows a promising activity in restoring cytarabine sensitivity in cytarabine resistant ALL patients. Therefore, the present study investigates the effects of 5-azacytidine, when applied before standard chemotherapy for AML.

3.1.1 Benefit / risk ratio

Patients eligible for this trial have a risk of treatment failure with standard chemotherapy of about 80% at two years. Treatment failure is an acutely life-threatening event for these patients. Included in these numbers are disease-related events (refractory disease, relapse) and treatment-related mortality that has to be expected to reach up to 10% in this patient group. The risk of this trial is that addition of 5-azacytidine to the standard treatment increases treatment-related death due to extensive toxicity. However, as pointed out above, phase I / II trials indicate that 5-azacytidine can be administered safely in combination with cytotoxic drugs.

The possible benefit of the trial is to decrease the rate of treatment failure in the trial population. For a single patient, this could result in increased life-span, ideally without evidence of leukemia. Given the extensively high rate of treatment failure despite the high toxicity of the currently available standard therapy, the medical need for novel, rational therapeutic approaches for this patient population is overwhelming. As pointed out above, the molecular rationale for the therapeutic administration of 5-

azacytidine in AML is well founded. Thus, the benefit/risk ratio for a patient to participate in this trial appears favorable.

3.2 Study objectives

3.2.1 Primary

- to compare the median Event Free Survival (EFS) of all AML patients between the 5-azacytidine and the control group

3.2.2 Secondary

- to compare the median Event Free Survival (EFS) of AML patients with different cytogenetic and molecular risk groups[1]
- to compare the median Overall Survival (OS) of all AML patients between the 5-azacytidine and the control group
- to compare the median Overall Survival (OS) of AML patients with different cytogenetic and molecular risk groups[1]
- to compare Relapse Free Survival (RFS) of AML patients between the 5-azacytidine and the control group
- to compare the rate of early response after the first induction cycle between the 5-azacytidine and the control group
- to compare the Complete Remission (CR) rate of the 5-azacytidine with the control group
- to compare the CR rate of AML patients with different cytogenetic and molecular risk groups[1]
- to compare the rate of molecular remissions of the 5-azacytidine with the control group
- to compare the toxicity of the 5-azacytidine and the control treatment
- to compare the evidence of minimal residual disease of all AML patients between the 5-azacytidine and the control group after induction therapy and in the course of the first remission
- to compare the development of biomarkers indicating the course of disease, including genetic, epigenetic, transcriptional and protein markers as well as indicators of neo-angiogenesis in leukemic blasts, bone marrow, peripheral blood cells, serum and plasma
- to compare the global methylation pattern and the methylation of selected gene promoters in the bone marrow and peripheral blood cells between the 5-azacytidine and the control group at different time points

---

[1] cytogenetic risk groups are defined as:
- cytogenetic good risk: t(8;21) or inv(16) without additional high-risk defining chromosomal aberrations
- cytogenetic intermediate risk: normal karyotype or chromosomal aberrations neither fulfilling good risk nor bad risk definitions
- cytogenetic high risk: t(3;3), -5, del(5q), t(6;9), t(6;11), -7, del(7q), +11, t(11;19)(q23;p13.1), abnormalities of 12p, +13, -17, del(17q), -18, -20, del(20q), complex karyotype (3 or more chromosomal aberrations)

within the intermediate group with normal karyotype, molecular risk groups are:
- molecular good risk: mutation of NPM and no Flt3-ITD
- molecular intermediate risk: all other combinations of NPM1 and Flt3 mutational status

- to evaluate the predictive value of changes of the methylation pattern for response in the 5-azacytidine group

3.3 Study design

This is a prospective, controlled, randomized, open, multi-center phase II study with parallel group design and fixed sample size and with a preceding dose finding run-in period. Patients older than 60 years (≥ 61 y) with newly diagnosed AML will receive standard induction and consolidation chemotherapy either preceded by 5-azacytidine (Arm A; study arm) or alone (Arm B; control arm). Patients of Arm A only will additionally receive a maintenance therapy with 5-azacytidine for one year after the start of induction therapy. For further details see chapter 5.

4 SELECTION, RANDOMIZATION AND WITHDRAWAL OF PATIENTS

4.1 Number of patients

According to the power calculations given in section 8.1, n = 216 patients will be included in the controlled phase of the study with an equal allocation rate to both treatment arms.

4.2 Admission criteria

4.2.1 Inclusion criteria

- Patients with newly diagnosed AML (except APL) according to the FAB or WHO classification, including AML evolving from MDS or other hematological diseases and AML after previous cytotoxic therapy or radiation (secondary AML).
- Bone marrow aspirate or biopsy must contain ≥ 20% blasts of all nucleated cells or differential blood count must contain ≥ 20% blasts. In AML FAB M6 ≥ 30% of non-erythroid cells in the bone marrow must be leukemic blasts. In AML defined by cytogenetic aberrations the proportion of blasts may be < 20%.
- Age ≥ 61 years
- Informed consent, personally signed and dated to participate in the study
- Male patients enrolled in this trial must use adequate barrier birth control measures during the course of the 5-azacytidine treatment and for at least 3 months after the last administration of 5-azacytidine.

4.2.2 Exclusion criteria

- Patients who are not eligible for standard chemotherapy as described in chapter 5.2 and 5.3

- Hyperleukocytosis (leukocytes > 20,000/µl) at study entry. These patients should be treated with hydroxyurea or receive leukocytapheresis treatment (if leukocytes > 100,000/µl) according to routine practice and entered into the study when leukocyte counts below 20,000/µl are reached. This applies only for the controlled part of the study.
- Patients with initial hyperleukocytosis above 20,000/µl can only be enrolled into the controlled part of the study, but not in the run-in dose finding part.
- Known central nervous system manifestation of AML
- Cardiac Disease: Heart failure NYHA class 3 or 4; unstable coronary artery disease (MI more than 6 months prior to study entry is permitted); serious cardiac ventricular arrhythmias requiring anti-arrhythmic therapy (beta blockers or digoxin are permitted)
- Chronically impaired renal function (creatinin clearance < 30 ml / min)
- Inadequate liver function (ALT and AST ≥ 2.5 x ULN) if not caused by leukemic infiltration
- Total bilirubin ≥ 1.5 x ULN if not caused by leukemic infiltration
- Known HIV and/or hepatitis C infection
- Evidence or history of severe non-leukemia associated bleeding diathesis or coagulopathy
- Evidence or recent history of CNS disease, including primary or metastatic brain tumors, seizure disorders
- Uncontrolled active infection
- Concurrent malignancies other than AML with an estimated life expectancy of less than two years
- History of organ allograft
- Hypersensitivity to cytarabine (not including drug fever or exanthema), daunorubicin, azacytidine or mannitol
- Previous treatment of AML except hydoxyurea and up to 2 days of ≤100 $mg/m^2/d$ cytarabine
- Previous therapy with 5-azacytidine (i.e. for an antecedent myelodysplastic syndrome)
- Patients with investigational drug therapy outside of this trial during or within 4 weeks of study entry should be discussed with the study office whether study participation is possible
- Any severe concomitant condition, which makes it undesirable for the patient to participate in the study or which could jeopardize compliance with the protocol

4.3 Registration, randomization and stratification

4.3.1 Registration

Registration and randomization (including out of office hours, weekends and holidays) will be performed via the AML-AZA-randomization-hotline at the Universitätsklinikum Münster, phone: 0251/83-44805.

ZZZ – NNN

ZZZ = three numbers for the Center

NNN = Unique patient identification number (continuously increasing number)

randomization: Arm A or B

Details for registration are:

- registration number (see below), sex and date of birth
- Diagnosis (according to the FAB-classification)
- Study center, name of the physician, phone and fax number of the center

4.3.2 Stratification and Randomization Procedure

The randomization lists will be deposited at the IMIB (Institut für Medizinische Informatik und Biomathemathik) at the Universitätsklinikum Münster**.** After registration the randomization result will be reported to the trial site via fax response on the registration form (Patientenmeldebogen). The registration number and the randomization result will be noted in the patient file and in the trial documentation forms (CRF).

4.4 Withdrawal of patients

A patient is free to withdraw from the study at any time for any reason without prejudice to his/her future medical care by the physician or at the institution. The Investigator may also withdraw the patient at any time in the interest of the patient`s safety. The primary reason for withdrawal must be recorded in the patient’s medical record and on the withdrawal form in the Case Report Form (CRF).

5 STUDY, STANDARD AND CONCOMITANT TREATMENTS

5.1 Investigational product

5-azacytidine is a ring analog of the pyrimidine nucleoside cytidine. In dosages approved for the treatment of myelodysplastic syndromes it inhibits the methylating enzyme DNA methyltransferase leading to a demethylation of CpG islands within gene promoters and the reversal of aberrant gene silencing. For full details of the pre-clinical and clinical information please refer to the summary of product characteristics and the investigators brochure.

5.1.1 Relevant physical, chemical and pharmaceutical properties

5-azacytidine is unstable in aqueous solutions. Therefore, the product is provided as a lyophilized powder, which requires reconstitution immediately prior to use. The drug product for supply of clinical trials is a vial with 100 mg 5-azacytidine as lyophilisated powder.

5.1.2 Instructions for storage and handling

The storage temperature of the vial containing the lyophilized substance 5-azacytidine should not exceed 30°C. The unreconstituted vials should be stored at 25°C (excursions permitted to 15-30°C). The lyophilized substance may only be used until the expiration date provided on the vial.

For intravenous infusion, the lyophilized powder will be reconstituted by suspension in 4 cc distilled water immediately before dilution. This reconstituted solution will then be diluted into 0.9% sodium chloride to an end volume of 50 to 500 cc. Administration of azacytidine diluted for IV administration must be completed within one hour of reconstitution of the azacytidine vial. No light protection is required during the infusion and the solution is compatible with PVC-containing infusion material.

For subcutaneous injection, the lyophilized powder will be reconstituted by suspension in 4 cc distilled water immediately before use. The suspension will be injected subcutaneously underneath the skin in the abdominal region, thigh or upper arm. For subcutaneous dosing, injection sites have to alternate between the body regions each time. Before injection, the skin at the injection site has to be thoroughly desinfected and even drug suspension has to be assured by gentle rolling of the injection syringe between both hands immediately before injection. New injections should be given at least one inch from an old site and never into hematomatous, dolent, erythematous or indurated skin. Excess suspension not required for the current injection will be discarded. If a dose in excess of 100 mg (4 mL) is required, the above step of preparation of the suspension is to be repeated. In such circumstances the dose should be equally divided into 2 syringes and injected into 2 separate sites.

Subcutaneous injections will be performed by nurses or physicians. After sufficient training, subcutaneous injections can be performed by the patients and / or his / her relatives.

5.1.3 Interactions with concomitant medications

Pharmacokinetic studies about the interaction between 5-azacytidine and other drugs have not been performed to date. *In vitro*, no induction or inhibition of CYP-enzymes could be detected by 5-azacytidine.

5.1.4 Drug accountability

The drug will be distributed to the center pharmacies or to the responsible investigator in the center in vials containing an approximately 1 month drug supply (10 vials of 100 mg 5-azacytidine each).

The center is responsible for ordering more supply at least three working days ahead of time.

Each center has to document in a list, when which drug was given to whom. This list has to contain the date, the patients name, the batch number, the use-by date and the number of vials dispensed.

The person applying the drug has to document the drug application and - in case of a failure to administer the medication - a short statement, why medication was not applied, in a standardized diary.

5.2 Standard cytotoxic therapy

Since the standard cytotoxic treatment used in this protocol is approved and belongs to the standard of care of the population included in this study, the standard cytotoxic treatment is NOT an investigational product in this study.

5.2.1 Cytarabine (AraC)

Cytarabine belongs to a group of chemotherapeutic agents called antimetabolites. Although the mechanism of action is not completely understood, it appears that cytarabine acts through the inhibition of DNA polymerase. A limited, but significant, incorporation of cytarabine into both DNA and RNA has also been reported.

Cytarabine is not active orally. It may be given by intravenous infusion or injection, subcutaneously, or into the liquor space. When large intravenous doses are given quickly, patients are frequently nauseated and may vomit for several hours after injection. This problem tends to be less severe when the drug is infused.

A cytarabine syndrome has been reported to occur in patients who received cytarabine. It is characterized by fever, myalgia, bone pain, occasionally chest pain, maculopapular rash, conjunctivitis and malaise. It usually occurs 6-12 hours following drug administration. Corticosteroids have been shown to be beneficial in treating or preventing this syndrome. If the symptoms of the syndrome are deemed treatable, corticosteroids should be considered as well as continuation of therapy with cytarabine.

Like other cytotoxic drugs, cytarabine may induce anemia, leukopenia, thrombocytopenia and hyperuricemia secondary to rapid lysis of neoplastic cells.

Acute pancreatitis has been reported to occur in patients being treated with cytarabine who have had prior treatment with L-asparaginase.

Severe and at times fatal CNS, GI and pulmonary toxicity has been reported following high dose schedules of cytarabine. These reactions include reversible corneal toxicity and hemorrhagic conjunctivitis, which may be prevented or diminished by prophylaxis with a local corticosteroid eye drop; cerebral and cerebellar dysfunction, including personality changes, somnolence and coma, usually reversible; severe gastrointestinal ulceration, pulmonary edema, liver damage with hyperbilirubinemia. Rarely, severe skin rash, leading to desquamation has been reported.

For full details of the drug information please refer to the "Fachinformationsverzeichnis Deutschland" in its latest version.

5.2.2 Daunorubicin

Daunorubicin has antimitotic and cytotoxic activity through a number of proposed mechanisms of action. It forms complexes with DNA by intercalation between base pairs. It inhibits topoisomerase II activity by stabilizing the DNA-topoisomerase II complex, preventing the religation portion of the ligation-religation reaction that topoisomerase II catalyzes. Single strand and double strand DNA breaks result. Daunorubicin may also inhibit polymerase activity, affect regulation of gene expression, and produce free radical damage to DNA.

Daunorubicin must be given into a rapidly flowing intravenous infusion. It must *never* be given by the intramuscular or subcutaneous route. Severe local tissue necrosis will occur if there is extravasation during administration.

Myocardial toxicity manifested in its most severe form by potentially fatal congestive heart failure may occur either during therapy or months to years after termination of therapy. The incidence of myocardial toxicity increases after a total cumulative dose exceeding 400 to 550 mg/m^2 in adults.

Severe myelosuppression occurs when used in therapeutic doses; this may lead to infection or hemorrhage.

Dosage should be reduced in patients with impaired hepatic or renal function.

Daunorubicin may transiently impart a red coloration to the urine after administration, and patients should be advised to expect this.

For full details of the drug information please refer to the "Fachinformationsverzeichnis Deutschland" in its latest version.

5.3 Treatment

5.3.1 Dose finding for 5-azacytidine

As a safety step for the combination therapy, the trial is preceded by a run-in part. Based on acceptable safety data for 5-azacytidine, cytarabine and daunorubicin each used in combination with other cytotoxic agents, there will be a rapid run-in dosing scheme in order to exclude potential toxicities of the combination therapy.

Patients in this part of the study will be randomized between dose level 1 (37.5 mg/m^2/day, n=6) and dose level 2 (75 mg/m^2/day, n=6). Patients will receive one or two induction therapy cycles with cytarabine and daunorubicin as described under 5.3.2, preceded by 5-azacytidine 37.5 mg/m^2/day (dose level 1) or 75 mg/m^2/day (dose level 2) as an intravenous infusion over 15 - 30 minutes once daily on days (-5) to (-1) before start of each induction therapy. All toxicity during induction therapy will be graded according to the Common Toxicity Criteria for Adverse Events v. 3.0 (CTCAE). Dose limiting toxicity is defined here as prolonged pancytopenia ≥ 42 days from start of last cytotoxic therapy. Patients with leukemic blast persistence in the bone marrow (day 15) will receive a second course of therapy (azacytidine, cytarabine, daunorubicin). All patients who achieve a complete remission after one or two cycles of induction therapy will receive 2 cycles of consolidation therapy as described under 5.3.3, preceded by 5-azacytidine 37.5 mg/m^2/day (dose level 1) or 75 mg/m^2/day (dose level 2) as a subcutaneous injection on days (-5) to (-1) before start of each consolidation therapy. Patients in the run-in part will NOT receive 5-azacytidine maintenance treatment. As extensive safety data is available for patients with myelodysplastic syndrome treated with 5-azacytidine monotherapy in the dosage used in the maintenance part of this study, the dosage of 5-azacytidine in the maintenance part will NOT be evaluated prior to start of the controlled part of the study. Within the dose finding phase, only patients with white blood cell counts below 20,000 /µl can be enrolled at the start of therapy.

If a patient enrolled in the dose finding phase develops a hyperleukocytosis > 50,000/µl within the 5-azacytidine period preceding the first induction therapy, the treatment with 5-azacytidine will be terminated (for this induction therapy cycle) and induction therapy will be started (see 5.3.2.2). This patient will be replaced by another patient at the same dose level if this patient develops no DLT[2].

If < 2 out of 6 patients experience DLT[2] at dose level 2, dose level 2 will be chosen for 5-azacytidine during the chemotherapy phase. If ≥ 2 of 6 patients experience DLT at dose level 2 and < 2 out of 6 patients experience DLT at dose level 1, dose level 1 will be chosen for 5-azacytidine during the chemotherapy phase.

If ≥ 2 of 6 patients experience DLT at dose level 2 and ≥ 2 out of 6 patients experience DLT at dose level 1 during the chemotherapy phase, dose level 0 will be evaluated during induction therapy. Only

[2] Dose limiting toxicity is defined here as prolonged pancytopenia ≥ 42 days from start of last cytotoxic therapy.

in the latter case further patients (n=6) will receive one or two induction therapy cycles with cytarabine and daunorubicin as described under 5.3.2, preceded by 5-azacytidine 18 mg/m^2/day as an intravenous infusion over 15 to 30 minutes on days (-5) to (-1) before start of each induction therapy. All patients who achieve a complete remission after one or two cycles of induction therapy will receive 2 cycles of consolidation therapy as described under 5.3.3, preceded by 5-azacytidine 18 mg/m^2/day as a subcutaneous injection on days (-5) to (-1) before start of each consolidation therapy.

If < 2 out of this 6 patients at dose level 0 have DLT during the induction treatment, dose level 0 will be chosen for 5-azacytidine during induction therapy. If ≥ 2 of 6 patients experience DLT at dose level 0 during the induction therapy, no 5-azacytidine will be applied during the induction treatment.

5.3.1.1 Scheme of the run-in part:

**Induction**		**Consolidation**	
**1. cycle**	**(2. cycle)** ***at blast persistence***	**1. cycle**	**2. cycle**
**7+3**	**(7+3)**	**HiDAC**	**HiDAC**
x	x	x	x

+ 5-azacytidine days (-5) – (-1) preceding the cycles*

x: dose level x for 5-azacytidine during chemotherapy

Note: For the purpose of clarity, the daily dose of 5-azacytidine during both induction and consolidation therapy in the controlled part of this protocol is described as “75 mg/m^2/day”. However, if the safety committee decides otherwise, the correct dose of 5-azacytidine in each part of the protocol (i.e. “37.5 mg/m^2/day during chemotherapy, 75 mg/m^2/day during maintenance therapy”) will be amended to the protocol for the controlled phase prior to start of the randomization (chemotherapy phase).

5.3.2 Controlled phase - Induction therapy

5.3.2.1 Arm A (Study arm)

AraC	100 mg/m2/day	continuous infusion over 24 hours	Days 1-7
Daunorubicin	45 mg/m2/day	infusion over 1-2 hours	days 3, 4 and 5
5-azacytidine	75 mg/m2/day	intravenous infusion over 15 to 30 minutes	days (-5) to (-1)

Patients with hyperleukocytosis > 20,000/µl can be treated with hydroxyurea or receive preceding leukocytapheresis treatment (if leukocytes > 100 000/µl) to lower leukocyte levels below 20,000/µl according to standard practice. When leukocyte counts below 20,000/µl are reached the patient can be enrolled into the study and can be randomized at this point. 5-azacytidine treatment can be started 24 h after the last dose of hydroxyurea. In patients with rapid kinetics whose leukocytes increase above 50,000 during 5-azacytidine treatment, 5-azacytidine treatment will be stopped for the induction cycle and standard chemotherapy is started the next day after the last 5-azacytidine infusion. In these patients, the other therapy cycles are applied as scheduled and include 5-azacytidine treatment.

If there are more than 5% blasts in the bone marrow (aspirate) on day 15, patients will receive a second (identical) course of induction therapy. 5-azacytidine treatment will be administered for five days before the second induction course and can be started on day 17. Cytarabine will be started on day 22. In case 5-azacytidine treatment starts later than day 17, the chemotherapy is postponed accordingly.

It is allowed to postpone the second course in case of an uncontrolled infection or other transitory contraindications against chemotherapy. No additional 5-azacytidine in addition to the 5 day-periods preceding the chemotherapy should be applied during the chemotherapy phase. This also applies in case of a delay between the completion of the 5-azacytidine and the onset of the following chemotherapy course.

If for any reason IV application of 5-azacytidine is no option at this point, patients enrolled in the controlled part of the induction therapy of the trial may be treated via subcutaneous azacytidine administration. However, IV application of the study medication is preferable.

5.3.2.2 Arm B (Standard cytotoxic therapy)

AraC	100 mg/m2/day	continuous infusion over 24 hours	Days 1-7
Daunorubicin	45 mg/m2/day	infusion over 1-2 hours	days 3, 4 and 5

If there are more than 5% blasts in the bone marrow (aspirate) on day 15, patients will receive a second (identical) course of induction therapy beginning on day 22.

It is allowed to postpone the second course if the patient has an uncontrolled infection or other transitory contraindications against chemotherapy.

5.3.3 Consolidation therapy

All patients in CR or in incomplete CR with platelets > 70,000/µl and neutrophils > 1,000/µl after induction therapy should receive two courses of high-dose cytarabine consolidation therapy not earlier than one week after attaining CR or incomplete CR with platelets > 70,000/µl and neutrophils > 1,000/µl. If, in the opinion of the treating physician, the patient's condition does not allow for consolidation therapy, consolidation therapy can be postponed for up to 4 weeks after a CR or incomplete CR with platelets > 70,000/µl and neutrophils > 1,000/µl is reached. If the patient is still considered not suitable for consolidation therapy after these 4 weeks, the consolidation therapy will be omitted and the patient will proceed to maintenance therapy or observation as described in 5.3.4

Criteria for the beginning of each consolidation course: Platelets > 70,000/µl and neutrophils > 1,000/µl.

5.3.3.1 Arm A (Study arm)

AraC	1g/m2 2 x/day	infusion over 3 hours	days 1, 3 and 5
5-azacytidine	75 mg/m2/day	subcutaneous injection	days (-5) to (-1)

The second course will start at least 1 week after regeneration of peripheral blood counts (platelets ≥ 70,000/µl, neutrophils ≥ 1,000/µl), but not earlier than day 28[3] of the first cycle. 5-azacytidine will be added to each cycle of the consolidation therapy. No additional 5-azacytidine should be administered between the consolidation therapy cycles. If for any reason subcutaneous application of 5-azacytidine

is no option at this point, patients enrolled in this phase of the trial may for exception be treated via IV administration. However, subcutaneous application of the study medication is preferable.

5.3.3.2 Arm B (Standard cytotoxic therapy)

AraC	1g/m2 2 x/day	infusion over 3 hours	days 1,3 and 5

The second course will start at least 1 week after regeneration of peripheral blood counts (platelets ≥ 70,000/µl, neutrophils ≥ 1,000/µl), but not earlier than day 28[3] of the first cycle.

5.3.4 Maintenance therapy

5.3.4.1 Arm A (Study arm)

All patients in CR or incomplete CR with platelets > 70,000/µl and neutrophils > 1,000/µl after consolidation therapy will receive maintenance therapy with 5-azacytidine 75 mg/m^2/day s.c. days 1-5 on a 28-day cycle. Maintenance therapy will start at least 1 week after regeneration of peripheral blood counts (platelets ≥ 70,000/µl, neutrophils ≥ 1,000/µl), but not earlier than day 23 of the last chemotherapy cycle. In addition, patients in CR or incomplete CR with platelets > 70,000/µl and neutrophils > 1,000/µl after induction therapy which are considered not suitable for consolidation therapy ≥ 4 weeks after achievement of CR / incomplete CR will proceed to maintenance therapy. Maintenance therapy will be administered until one year after start of induction therapy.

5.3.4.2 Arm B (Standard cytotoxic therapy)

Patients will not receive specific maintenance therapy but will be followed similar to patients treated in Arm A.

[3] the interval between two cycles is defined as the interval between the first administration of AraC in each cycle

5.3.5 Dose modification and delays of 5-azacytidine

The modifications of 5-azacytidine will follow the following pre-defined dose levels:

Dose level 3	100 mg/m^2
Dose level 2 (starting dose):	75 mg/m^2
Dose level 1:	37.5 mg/m^2
Dose level 0:	18 mg/m^2

5.3.5.1 General dose modifications

If a dose reduction below 18 mg/m^2 is required, the patient should be discontinued from the study treatment. Escalation to dose level 3 will be performed only if no toxicity occurred after two consecutive cycles of 5-azacytidine maintenance at dose level 2. After resolution of the adverse event the dose may be re-escalated from dose level 1 or 0 to the standard dose at the discretion of the investigator. As a general rule, grade 3 toxicity should be followed by permanent dose reduction of 5-azacytidine during that therapy phase (chemotherapy phase or maintenance phase). If a grade 3 toxicity during the chemotherapy phase resolves after dose reduction of 5-azacytidine, the dosage of 5-azacytidine may be re-escalated after two cycles of maintenance therapy if no toxicity except nausea and vomiting occurs after the first two cycles of maintenance therapy. Resolution of an adverse event is defined as disappearance or reduction of the adverse event to < grade 3 toxicity. For patients with grade 2 or greater toxicities present at baseline resolution to at least baseline levels will apply.

5.3.5.2 Dose modifications for hematological toxicity

Hematological toxicities during the period of chemotherapy and the expected subsequent myelosuppression will only be considered relevant if a grade IV neutropenia or thrombopenia persists ≥ 42 days after start (day 1) of the last chemotherapy cycle. After a relapse is excluded by bone marrow aspiration between day 35 and 42, therapy with 5-azacytidine will be delayed until hematological recovery (neutrophils > 1,000/µl, platelets > 70,000/µl) and the dose will be reduced by one dose level for all subsequent chemotherapy cycles and the first two maintenance cycles. If no toxicity except nausea and vomiting occurs after the first two cycles of 5-azacytidine maintenance at the reduced dose level, the dose level may be re-escalated by one dose level for the next maintenance cycles.

However, different criteria will apply after hematopoietic recovery from the last chemotherapy course during the subsequent maintenance therapy.

Table 1-1 illustrates dose modifications and delays for hematological toxicities during the maintenance therapy:

Table 1-1: Hematological Criteria for Dose Delay and Dose Modification of 5-azacytidine during maintenance therapy		
Grade	Dose Delay	Dose Modification
Grade 0-2	Treat on time	No Change [b]
Grade 3	Treat on time	DECREASE one dose level [c]
Grade 4	DELAY [a] until ≤ Grade 2	DECREASE one dose level [c]

a. If no recovery after 30 days delay, treatment will be discontinued
b. If no toxicities except nausea / vomiting occur during two consecutive maintenance cycles, the dose will be increased by one dose level to a maximum dose of 100 mg/m^2
c. If a dose reduction below 18 mg/m^2 is required, treatment will be discontinued

5.3.5.3 Dose modifications for non-hematological toxicity

Table 1-2 summarizes the recommendations for dose delays and modifications for all non-hematological adverse events.

Table 1-2: Non-hematological Criteria for Dose Delay and Dose Modification of 5-azacytidine		
Grade	Dose Delay	Dose Modification
Grade 0-2	Treat on time	No Change [b]
Grade 3	DELAY [a] until ≤ Grade 2	DECREASE one dose level [c]
Grade 4	OFF protocol therapy	OFF protocol therapy

a. If no recovery after 30 days delay, treatment will be discontinued
b. If no toxicities except nausea / vomiting occur during two consecutive maintenance cycles, by one dose level to a maximum dose of 100 mg/m^2
c. If a dose reduction below 18 mg/m^2 is required, treatment will be discontinued

These recommendations pertain, if the adverse event is considered to be related to 5-azacytidine, but not to the chemotherapy.

5.4 Concomitant medications / therapy

All appropriate palliative and supportive care for disease-related symptoms will be provided to all patients. Sufficient anti-emetic prophylaxis is required prior to the administration of standard chemotherapy and 5-azacytidine according to the study centers' standard procedures. Symptomatic treatments should be used as needed and may be given as prophylactics in subsequent cycles.

Anti-microbial prophylaxis including antifungal prophylaxis in the presence of myelosupression should be given according to the study centers' standard procedures.

G-CSF will be given at a dose or doses in accordance with the study centers' standard practice regarding the use of growth factors. It is also to the discretion of the participating centers, which type of G-CSF preparation they choose to treat their patients. Available preparations are filgastrim (Neupogen®), lenogastrim (Granocyte®) and the pegylated form pegfilgastrim (Neulasta®).

Hydroxyurea may be used according to the centers` standard practice to decrease initial hyperleukocytosis (leukocytes ≥ 20,000/µl) before starting the first induction therapy cycle. (This does not apply to patients enrolled in the run-in phase. In this case, patients with hyperleukocytosis above 20,000/µl are excluded from study participation).

Leukocytapheresis is not contraindicated in this study and may be an option to decrease elevated leukocyte counts (if leukocytes > 100,000/µl).

6 STUDY PROCEDURES

6.1 Study evaluations

Most procedures for patient evaluation that are listed below are considered by the investigators to be necessary according to the standard of good clinical practice for the diagnosis and treatment monitoring of AML patients.

6.1.1 Evaluations before treatment

- Complete blood count (CBC) with differential and platelets
- Serum chemistries: electrolytes, creatinine, urea, uric acid, bilirubin, AP, AST and / or ALT, LDH, lipase, phosphate, bicarbonate, creatinine kinase.
- Coagulation: PTT, quick, fibrinogen, ATIII
- Urine analysis
- Blood type
- Full history and clinical examination
- Vital signs
- Body height and body weight
- Performance status (ECOG)
- ECG
- Echocardiography or MUGA scan[4]
- Ultrasound of the abdomen
- Bone marrow aspirate and biopsy:

  - Cytogenetics

  - Cytomorphological examination incl. Cytochemistry

  - Immunophenotyping

  - Molecular genetic analyses for the presence of Flt3-ITD, genomic ratio of the presence of Flt3-ITD vs. Flt3-WT, for nucleophosmin mutations, for the presence of Bcr-Abl, PML-RARα, AML1-ETO and CBFß-MYH11

  - Asservation of vital bone marrow cells (Ficoll-treated bone marrow), RNA, DNA and protein lysates, serum and plasma in a central tissue repository[5]

  - global methylation status, methylation status of known methylated tumor suppressor genes[5]

---

[4] The same method for determination of the left ventricular function should be used for each patient throughout the study participation

[5] These analyses will be performed for exploratory research purposes. They are optional and will not be part of the medical report of this trial

6.1.2 Evaluations during treatment

(for full details about evaluations please refer to 6.2)

- Bone marrow aspirate
- Asservation of vital bone marrow cells (Ficoll-treated bone marrow), bone marrow RNA, DNA and protein lysates, serum and plasma in a central tissue repository[5].
- Control of CBC (with differential and platelets), coagulation and serum chemistries (including creatinine kinase, creatinine, bicarbonate and phosphate)
- Performance status
- Physical examination
- Body weight
- Diagnostic procedures i.e. in case of severe infections will follow standard procedures
- Evaluations performed before the first course of induction therapy will be repeated prior to each following course if necessary in the investigator's opinion
- ECG and echocardiography / MUGA scan[4] as often as necessary in the investigator's opinion
- global methylation status, methylation status of known methylated tumor suppressor genes[5]

6.1.3 Evaluations at the end of maintenance therapy

(for full details about evaluations please refer to 6.2)

- Performance status
- Physical examination
- Vital signs
- CBC (with differential and platelets), coagulation and serum chemistries (including creatinine kinase, creatinine, bicarbonate and phosphate)
- Urine pH
- Bone marrow aspirate
- Asservation of vital bone marrow cells (Ficoll-treated bone marrow), bone marrow RNA, DNA and protein lysates, serum and plasma in a central tissue repository[5]
- global methylation status, methylation status of known methylated tumor suppressor genes[5]

---

[4] The same method for determination of the left ventricular function should be used for each patient throughout the study participation

[5] These analyses will be performed for exploratory research purposes. They are optional and will not be part of the medical report of this trial

6.1.4 Evaluations in case of relapse

- CBC (with differential and platelets)
- Bone marrow aspirate
- Asservation of vital bone marrow cells (Ficoll-treated bone marrow), bone marrow RNA, DNA and protein lysates, serum and plasma in a central tissue repository[5]
- global methylation status, methylation status of known methylated tumor suppressor genes[5]

6.1.5 Evaluations during follow up (standard routine parameters)

In case of a relapse, a study exclusion or a refusal of the informed consent, patients should be followed until one year after the start of the induction therapy. These follow-up evaluations include:

- Bone marrow aspirate in case of suspected relapse
- CBC with differential monthly
- Performance status monthly

6.2 Schedule of evaluations

	Within 10 days prior to the first course of induction therapy	Day 1 of the first induction therapy before the first administration of cytarabine	During and between induction therapy until hematological recovery	d 15	Before the second course of induction therapy	d 35 after last course of induction	Before the first course of consolidation therapy
Informed consent	X						
Medical history	X						
Performance status and physical examination	X				X		X
Vital Signs (blood pressure and pulse)[a]	X				X		X
Body weight and height	X				X		X
CBC (with differential and platelets)[a]	X	X[c]	Once weekly		X	X	X
Chemistry, including liver function, creatinine, coagulation[a] and bicarbonate	X		Once weekly		X		X
Urinalysis[a]	X						
ECG and echocardiography / MUGA scan[ab]	X						

[5] These analyses will be performed for exploratory research purposes. They are optional and will not be part of the medical report of this trial

	Within 10 days prior to the first course of induction therapy	Day 1 of the first induction therapy before the first administration of cytarabine	During and between induction therapy until hematological recovery	d 15	Before the second course of induction therapy	d 35 after last course of induction	Before the first course of consolidation therapy
Ultrasound of the abdomen[a]	X						
Bone marrow aspirate	X			X			X
Bone marrow biopsy	X						
Bone marrow and blood samples for exploratory research	X			X			X
Methylation analysis	X[d]	X[d]		X[d]			X[d]

	Before the second course of consolidation therapy	Start of maintenance therapy	During maintenance therapy or observation	End of maintenance therapy	Follow –up: until one year after the first induction therapy	Suspected relapse (during study treatment or follow-up)
Informed consent						
Medical history						
Performance status and physical examination	X	X	monthly	X	X	
Vital Signs (blood pressure and pulse)[a]	X	X	monthly	X		
Body weight and height	X					
CBC (with differential and platelets)[a]	X	X	monthly	X	X	X[f]
Chemistry, including liver function and creatinine, , coagulation[a] and bicarbonate	X	X	monthly	X		
Urinalysis[a]						
ECG and echocardiography / MUGA scan[ab]						

Sonography of the abdomen[a]						
Bone marrow aspirate	X		X[e]	X	X	X[f]
Bone marrow biopsy						
Bone marrow and blood samples for exploratory research	X		X[e]	X	X	X[f]
Methylation analysis	X[d]		X[de]	X[d]	X[d]	X[df]

[a] In addition, these evaluations should be repeated as often as necessary in the opinion of the treating physician

[b] The same method for determination of the left ventricular function should be used for each patient throughout the study participation

[c] Material for differential CBC may be stored and analyzed on the next working day

[d] Methylation analysis on day 1 before the first induction therapy will be performed from peripheral blood only. For all other time points, methylation analysis will be performed from peripheral blood and bone marrow

[e] One month after begin of maintenance therapy or observation and then every 3 months or at suspected relapse

[f] For patients with suspected or confirmed relapse. For patients with a leukocytopenia < 1,000/µl or thrombocytopenia < 70,000/µl ≥ 35 days after start (day 1) of the last induction therapy or of a consolidation therapy cycle, these evaluations will be performed between day 35 and 42.

6.3 Bone marrow diagnostics including central diagnostics

The diagnosis of AML will be performed at the participating centers. For the central cytomorphological review, central bone marrow biopsy diagnosis, central cell repository and the centralized analyses of DNA methylation, bone marrow aspirate, bone marrow biopsy and peripheral blood of each patient have to be sent to Dresden, Frankfurt or Münster (see 6.3.5).

6.3.1 Cytomorphologic examination and Cytochemistry

Initial leukemia diagnostics and differentiation are based on the morphology of peripheral blood and bone marrow smears as well as on cytochemical examinations. If the bone marrow aspirate is not available, diagnosis will be based on the bone marrow biopsy or on peripheral blood.

A Pappenheim staining, a peroxidase reaction and an esterase reaction are required. Diagnosis and classification of the AML will be performed according to FAB criteria as well as according to the WHO classification. These analyses will be performed in the local participating centers. The central study offices participate in round robin tests within the scope of the network of excellence for acute and chronic leukemias and offer reference laboratory diagnostics for discussion of questionable cases. To be able to quickly assess the diagnostic material, bone marrow and peripheral blood smears are requested for all patients at diagnosis (for shipping modalities, see 6.3.5). For discussion of cytomorphology, the laboratories in Dresden, Frankfurt or Münster can be contacted.

6.3.2 Immunphenotyping

An immunophenotyping of the leukemia cells should be performed for all patients. These analyses are performed at the local study centers. The immunophenotyping should contain the antigen CD56 and should be performed according to the proposals by the network of excellence acute and chronic leukemias.

6.3.3 Cytogenetics

For each patient a cytogenetic examination has to be performed at diagnosis. Chromosomal G-banding will be performed. Fluorescent-in-situ-hybridizations will be performed if necessary. The cytogenetic examination will be organized by the local study centers.

Cytogenetic laboratories:

(Other laboratories may also be chosen.)

Dr. U. Pascheberg / W. Peter<br>Gemeinschaftspraxis für Laboratoriumsmed.<br>Dr. Eberhard und Partner<br>Brauhausstr. 4<br>44137 Dortmund<br><br>*Phone: 0231/9572-606*<br>*Fax: 0231/9572-636*	Dr. E. Krasemann<br>Labor Drs. med. Fenner<br>Abt. Humangenetik<br>Bergstr. 14<br>20095 Hamburg<br><br>*Phone: 040/30955-0*<br>*Fax: 040/30955-13*
Prof. Dr. Dr. H. Zankl / Dr. B. Thiele<br>Institut für Immunologie und Genetik<br>am Klinikum Kaiserslautern<br>Laborarztpraxis<br>Hellmut-Hartert-Str. 1<br>67613 Kaiserslautern<br><br>*Phone: 0631/316-700*<br>*Fax: 0631/316-7020 / 21*	Dr. rer. medic. B. Mohr<br>Universitätsklinikum Dresden<br>Med. Klinik und Poliklinik I<br>Haus 65 a<br>Fetscherstr. 74<br>01307 Dresden<br><br>*Phone: 0351/458-3377*<br>*Fax: 0351/458-4394*
Prof. Dr. Wieacker / Dr. S. Volpert<br>Institut für Humangenetik<br>Universitätsklinikum Münster<br>Vesaliusweg 12-14<br>48149 Münster<br><br>*Phone: 0251/83-55401*<br>*Fax: 0251/83-55431*	Prof. Dr. med. T. Haferlach / PD Dr. med. C. Haferlach<br>MLL Münchner Leukämielabor GmbH<br>Max-Lebsche-Platz 31<br>81377 München<br><br>*Phone: 089/990-17-0*<br>*Fax: 089/990-17-111*

6.3.4 Molecular genetic analyses

All molecular genetic analyses may be performed at the participating centers or at a laboratory of the investigators' choice.

Molecular genetic analysis has to include:

- determination of the presence or absence of Bcr-Abl, PML-RARα, AML-ETO, and CBFβ-MYH11 fusion transcripts as determined by qualitative or quantitative RT-PCR
- Nucleophosmin mutational status
- Determination of the presence or absence of Flt3-ITD and a quantification of the Flt3-ITD to Flt3-WT genomic ratio in case of the detection of Flt3-ITD

For central molecular analysis, 5 ml heparinized bone marrow can be sent to the laboratories in Dresden, Frankfurt or Münster (other laboratories may also be chosen).

6.3.5 Central bone marrow diagnostics and shipment modalities

Bone marrow (aspirate and biopsy) as well as peripheral blood for all central diagnostics (central cytomorphological review, bone marrow biopsy diagnosis, central cell repository and the centralized analyses of DNA methylation) should be sent to one of the following laboratories:

Universitätsklinikum<br>Carl Gustav Carus<br>Med. Klinik und Poliklinik I<br>Hämatologisches Labor Haus 65 a<br>Fetscherstr. 74<br>01307 Dresden<br><br>Phone: 0351/458-4251<br>Fax: 0351/458-4367<br><br>For information on results, contact:<br><br>Prof. Dr. C. Thiede<br>*Phone: 0351/458-5628*	Universitätsklinikum Münster<br>Medizinische Klinik A<br>Labor für Spezielle Hämatologie<br>(Ebene 05)<br>Albert-Schweitzer-Straße 33<br>48149 Münster<br><br>Phone: 0251/83-47608<br>Fax: 0251/83-47633<br><br>For information on results, contact:<br><br>Dr. Utz Krug<br>Email: Utz.Krug@ukmuenster.de<br><br>AML.Vidaza@uni-muenster.de<br><br>*Phone: 0251/835-2995*	Universitätsklinikum Frankfurt<br>Medizinische Klinik II<br>Labor für Molekulare Diagnostik<br>Haus 33, UG, Raum 6<br>Theodor-Stern-Kai 7<br>60590 Frankfurt<br><br>Phone: 069/6301-83044<br>Fax: 069/6301-83046<br><br>For information on results, contact:<br><br>Dr. med. C. Brandts<br>Email: brandts@em.uni-frankfurt.de<br>*Phone: 069/6301-7104*<br><br>Dr. med. P. Paschka<br>Email: paschka@em.uni-frankfurt.de<br>*Phone: 069/6301-5398*

6.3.5.1 Central bone marrow diagnostics at time of diagnosis:

For the central cytomorphological review, bone marrow biopsy diagnosis, central cell repository and the centralized analyses of DNA methylation, bone marrow aspirate, bone marrow biopsies and peripheral blood of each patient have to be sent to Dresden, Frankfurt or Münster at the time of diagnosis. All biopsies will then be transferred to Münster for analysis by Prof. Dr. G. Köhler at the Gerhard-Domagk-Institut für Pathologie, Domagkstr. 17, 48149 Münster, phone: 0251/83-55440. The diagnostic report of the bone marrow biopsy will be sent directly to the participating centers. There will be no charge for the bone marrow biopsy analysis and the fixation solution will be provided.

Analysis of the global methylation status and the methylation status of selected tumor suppressor genes will be performed retrospectively for exploratory research purposes.

For further investigations the remaining material will be cryo-preserved at the central study office. Upon consultation with the study group it is going to be available for further laboratory scientific investigations.

Requested material at time of diagnosis:

**Immediately at diagnosis, please collect for central diagnostics:**
3 x 5 ml heparinized bone marrow
1 x 5 ml bone marrow aspirate in EDTA
20 ml heparinized peripheral blood
at least 4 unstained bone marrow smears
2 unstained peripheral blood smears
bone marrow biopsy in provided fixation solution

Shipment is recommended from Monday through Thursday, if possible in the morning by express delivery or courier.

6.3.5.2 Central bone marrow diagnostics during treatment:

Please refer to 6.2: Schedule of evaluations.

Requested material during treatment:

**During study, please collect for central diagnostics**
3 x 5 ml heparinized bone marrow
1 x 5 ml bone marrow aspirate in EDTA
20 ml heparinized peripheral blood
at least 4 unstained bone marrow smears
2 unstained peripheral blood smears

Shipment is recommended from Monday through Thursday, if possible in the morning by express delivery or courier.

For the methylation analysis on day 1 of the first induction therapy, only 20 ml heparinized peripheral blood is requested.

6.4 Duration of the study

Accrual time: 24 months	Treatment / observation[a]: 12 months

[a] In case of a relapse / treatment failure, a study exclusion or a refusal of informed consent, patients will be followed up until one year after the start of the first induction cycle.

Assuming an accrual time of 24 months and a duration of treatment, observation or follow-up (of the last included patient) of approximately 12 months the duration of the study will be approximately 36 months.

6.5 End of study

The study will end one month after the end of the therapy, observation or follow-up period of the last patient.

6.6 Criteria for removal from study / premature end of study

6.6.1 Individual reasons (Criteria for removal of patients)

Patients will be removed from the trial for the following reasons:

- Drug-related toxicity
- Patient decision
- Incompliance

6.6.2 General reasons

The trial will be prematurely ended, if the safety committee recommends it for extensive toxicity in the 5-azacytidine treatment arm.

7 ADVERSE EXPERIENCES

7.1 Definitions

7.1.1 Adverse Event (AE)

Adverse event: Any untoward medical occurrence in a patient or clinical trial subject administered a medicinal product and which does not necessarily have a causal relationship with this treatment.

Comment: An adverse event (AE) can therefore be any unfavourable and unintended sign (including an abnormal laboratory finding), symptom, or disease temporally associated with the use of an investigational medicinal product, whether or not considered related to the investigational medicinal product.

Protocol-specific clarifications to this definition:

Symptoms of the disease under study should not be classified as AEs as long as they are within the normal day-to-day fluctuation or expected progression of the disease.

Abnormal laboratory values of creatinine kinase, creatinine and serum bicarbonate are adverse events of interest and have to be documented as AEs. The reporting of other abnormal laboratory values should be avoided unless they lead to clinical consequences that are not routine.

7.1.2 Adverse Reaction (AR)

Adverse reaction of an investigational medicinal product: All untoward and unintended responses to an investigational medicinal product related to any dose administered.

Comment: All adverse events judged by either the reporting investigator or the sponsor as having a reasonable causal relationship to a medicinal product qualify as adverse reactions. The expression reasonable causal relationship means to convey in general that there is evidence or argument to suggest a causal relationship.

7.1.3 Unexpected Adverse Reaction (UAR)

Unexpected adverse reaction: an adverse reaction, the nature, or severity of which is not consistent with the applicable product information.

Comments:

- When the outcome of the adverse reaction is not consistent with the applicable product information this adverse reaction should be considered as unexpected.
- Severity: The term "severe" is often used to describe the intensity (severity) of a specific event. This is not the same as "serious," which is based on patient/event outcome or action criteria.

Examples of UAR include:

- A more specific reaction than labeled ("acute renal failure" is a labeled AR, a new report of "interstitial nephritis" is more specific and therefore unexpected).
- An increase in the rate of occurrence of an expected AR, which is judged to be clinically important is considered as unexpected.

7.1.4 Definition of Serious Adverse Event (SAE) or Serious Adverse Reaction (SAR)

Serious adverse event or serious adverse reaction: any untoward medical occurrence or effect that at any dose

- results in death,
- is life-threatening,
- requires hospitalisation or prolongation of existing inpatients' hospitalization,
- results in persistent or significant disability or incapacity,
- is a congenital abnormality or birth defect.

Comments:

- All deaths including death to disease progression up to and including 42 days after the last protocol treatment including standard chemotherapy have to be reported immediately on an SAE form, although death is an outcome per definition, not an adverse event per se. The adverse event is the underlying event which led to death.
- Life-threatening in the definition of a serious adverse event or serious adverse reaction refers to an event in which the subject was at risk of death at the time of event; it does not refer to an event which hypothetically might have caused death if it was more severe.
- Hospitalization means overnight admission.
- Hospitalization without underlying adverse event (AE) is not an SAE. Examples are:
    - Hospitalization for protocol procedures e.g. chemotherapy
    - Elective hospitalization for a pre-existing condition (i.e. a condition other than the indication for the chemotherapy) that has not worsened
    - Admission to a rehabilitation centre or hospice
    - Hospitalization for social reasons (e.g. due to anxiety but otherwise treatable on an outpatient basis).
- Congenital abnormality or birth defect: Fathering a child under 5-azacytidine is reportable as SAE in order to identify and follow-up on outcome of pregnancy and on any congenital abnormalities. The report should be made as soon as the investigator gets knowledge of the

pregnancy. Follow-up of each pregnancy will be done using specific additional questionnaires supplied by the Safety Desk.

- Medical judgment should be exercised in deciding whether an adverse event/reaction is serious in other situations. Important adverse events/ reactions that are not immediately life-threatening or do not result in death or hospitalization but may jeopardize the subject or may require intervention to prevent one of the other outcomes listed in the definition above, may also be considered serious.
- A new malignancy is a medically important condition and always considered as serious adverse event according to this protocol.

Protocol-specific clarifications to this definition and exceptions for SAE reporting:

Leukemia-associated serious adverse events do not have to be reported as serious adverse events on this protocol. All deaths including death to disease progression have to be reported as SAE.

Myelosuppression, thrombocytopenia, anemia and associated complications are expected events during leukemia therapy and are part of the treatment success (marrow emptying of leukemia cells). Therefore, myelosuppression-associated complications such as fever, infections, bleeding, and related hospitalization will be reported on the adverse event pages of the CRF as an adverse event. In this context only prolonged myelosuppression, i.e. pancytopenia with marrow hypocellularity on day 42 or later from start of last cytotoxic therapy without evidence of leukemia, will require immediate reporting on an SAE form. This protocol-specific rule applies only for induction and consolidation courses, not during maintenance therapy.

7.1.5 Suspected Unexpected Serious Adverse Reaction (SUSAR)

A suspected unexpected serious adverse reaction (SUSAR) is a serious adverse reaction (SAR) that has been judged to be unexpected.

7.2 Period of observation, documentation and reporting

AEs and SAEs will be recorded from the time the informed consent is signed, up to and 42 days after the last protocol treatment including standard chemotherapy. AEs and SAEs for patients in arm B will be recorded up to one year after start of induction therapy or until end of trial participation, whichever happens earlier, but at least 42 days after last protocol treatment (equal period as those for patients in arm A receiving maintenance therapy). In case a patient leaves this protocol to receive another more intensive cytostatic therapy (that is for persistent or recurrent disease), the following modification applies: AEs and SAEs occurring within 42 days after last protocol treatment and after first administration (day 1) of the other, more intensive therapy have only to be recorded on the present protocol when the investigator suspects the event to be related to the protocol treatment.

All AEs, including SAEs, have to be recorded on the appropriate adverse event pages in the CRF.

If possible, a diagnosis rather than a list of signs, symptoms and laboratory abnormalities should be given.

All AEs, including SAE, must be followed up until the condition resolves or stabilizes.

All SAEs identified by the protocol as requiring immediate reporting must be reported on the SAE form and transmitted by fax to the Safety Desk within 1 business day of knowledge by the investigator. This applies regardless of severity (CTCAE 3.0 grade) and whether or not the SAE is considered related to the use of study drug by the investigator. Personal data have to be replaced by the trial patient number before forwarding any information.

Where possible, a diagnosis rather than a list of symptoms should be given. The investigator is responsible for assessment of seriousness, severity (CTCAE v 3.0) and causality of the SAE. The SAE form should be completed with as much information as possible. The investigator should not wait for full details before making the initial report.

Safety-Desk-Contact
Zentrum für Klinische Studien (ZKS) Münster<br>Von-Esmarch-Str. 62<br>48149 Münster<br>Phone: 0251 83 57109<br>Fax: 0251 83 57112<br>E-Mail:<br>mssd@ukmuenster.de

If the event is fatal or life threatening, the investigator must fax any relevant follow-up information of the reported SAE to the Safety Desk within additional 8 days. In case of death, a copy of the autopsy

protocol should be provided, if any. For SAEs, which are not fatal or life threatening, the investigator must fax follow-up information as soon as possible.

In case of death, the investigator has to supply sponsor, competent authority and Ethics Committee with all details requested.

The Co-ordinating Investigator will review each SAE again for seriousness and relatedness and assess each serious adverse reaction (SAR) for expectedness according to the Investigator's Brochure.

It is the duty of the Safety Desk to ensure that Ethics Committee, competent authority and participating investigators are informed of all suspected unexpected serious adverse reactions (SUSARs) and all other relevant safety information in accordance with legal requirements.

It is the duty of the Safety Desk to inform the marketing authorization holder involved according to stipulation.

The Co-ordinating Investigator is responsible for the ongoing safety evaluation of the trial. The Safety Desk will inform the Co-ordinating Investigator immediately about any relevant safety information coming to its knowledge as will the Co-ordinating Investigator inform the Safety Desk. In case of other safety relevant issues (besides SUSARs) which require expedited reporting, the Safety Desk will support the Co-ordinating Investigator in submitting an appropriate report in due time.

The Co-ordinating Investigator is responsible for providing the updated benefit-risk assessment of the trial for the Annual Safety Report (Part 1 of the report). The Safety Desk is responsible for preparing all other parts of the Annual Safety Report, finalizing it and submitting it to the competent authority and ethics committee in due time.

The Safety Desk will provide information for the Safety Committee as requested.

7.3 Warnings and Precautions

7.3.1 Investigational product (5-azacytidine)

7.3.1.1 Summary of known adverse drug reactions

Hypersensitivity and known adverse drug reactions to the investigational product 5-azacytidine may include:

The most frequent adverse events attributed to 5-azacytidine are nausea, vomiting and diarrhea. For this reason, antiemetic prophylaxis and therapy is required according to the study center's standard procedures.

Hematological toxicities are the second most common adverse event. Hematological grade IV toxicity is expected after induction and consolidation therapy and no dose modifications of 5-azacytidine apply during that phase, except for the case that the time to hematological regeneration from the last chemotherapy course exceeds 42 days (see 5.3.5).

Additional common adverse events of 5-azacytidine are local erythema and pain at the injection site and elevation of retention parameters, infrequently with renal failure and renal tubular acidosis.

A rare complication of a therapy with either 5-azacytidine or cytarabine is a rhabdomyolysis (13). No cases of rhabdomyolysis have been published in the limited number of patients treated with a combination of 5-azacytidine and cytarabine so far. Nevertheless, the levels of creatinine kinase will be regularly evaluated in patients participating in this study.

While there is no evidence that 5-azacytidine affects the ability to drive or operate machinery, patients experiencing vertigo or other symptoms that could interfere with their ability to drive a car or operate machinery should refrain from driving and operating machinery while under treatment with 5-azacytidine.

7.3.1.2 List of all adverse drug reactions reported in patients in multiple clinical studies in MedDRA coding:

System organ category	Preferred term	Frequency
very common >10% -100%; common >1% -10%; uncommon >0.1%-1%; rare < 0,1%		
Infections and infestations	Pneumonia	very common
	Upper respiratory tract infection	very common
	Pyrexia	Very common
	Herpes simplex	common
	Bacterial infection	common
Blood and lymphatic system disorders	Leukopenia	very common
	Anemia	very common
	Neutropenia	very common
	Febrile neutropenia	very common
	Thrombocytopenia	very common
	Lymphadenopathy	common
	Increased anemia	common
	Agranulocytosis	uncommon
Immune system disorders	Hypersensitivity reactions	common
Metabolism and nutrition disorders	Anorexia	very common
	Hypokalemia	very common
	Hypophosphatemia	very common
Psychiatric disorders	Restlessness	very common
	Anxiety	very common
	Depression	very common
	Insomnia	very common
	Asthenia	common
Nervous system disorders	Vertigo	very common
	Cephalgia	very common
	Peripheral sensory neuropathy (including dysaesthesia, hypaesthesia, hyperaesthesia)	common
	Intracranial hemorrhage	common
Vascular disorders	Petechial hemorrhage	very common
	Flush	common
	Hematoma	common
	Hypotension	common
Cardiac disorders	Heart murmurs	very common
	Tachycardia	common

Respiratory, thoracic and mediastinal disorders	Dyspnea	very common
	Coughing	very common
	Pharyngitis	very common
	Chest pain	very common
	Epistaxis	very common
	Productive coughing	very common
	Lung rhonchi	very common
	Rhinorrhoa	very common
	Rhonchi	common
	Wheeze	common
	Decreased breath sounds	common
	Pleural effusion	common
	Postnasal drip	common
	Atelectasis	common
	Worsening of dyspnea	common
	Sinusitis	common
	Hemoptysis	common
	Impaired nasal ventilation	common
Gastrointestinal disorders	Diarrhea	very common
	Nausea	very common
	Vomitus	very common
	Constipation	very common
	Abdominal pain	very common
	Abdominal pressure pain	very common
	Gastrointestinal tract bleeding	common
	Gingival bleeding	common
	Gingival petechial bleeding	common
	Unformed stool	common
	Stomatitis	common
	Dyspepsia	common
	Hemorrhoids	common
	Meteorism	common
	Dysphagia	common
	Enoral bleeding	common
	Tongue ulcers	common
	Perirectal abscess	uncommon
Skin and subcutaneous tissue disorders	Ecchymosis	very common
	Pallor	very common

	Dimple forming edema	very common
	Skin lesions	very common
	Hematoma at the injection site	very common
	Reaction at the injection site	very common
	Pruritus	very common
	Erythema	very common
	Increased sweating	very common
	Rash	very common
	Night sweating	common
	Cellulitis	common
	Pruritus at the injection site	common
	Rash with pruritus	common
	Urticaria	common
	Dry skin	common
	Granuloma at the injection site	common
	Hyperpigmentation at the injection site	common
	Swelling at the injection site	common
	Skin nodules	common
Musculoskeletal and connective tissue disorders	Arthralgia	very common
	Painful extremities	very common
	Backache	very common
	Contusion	very common
	Myalgia	very common
	Muscle cramps	common
	Rhabdomyolysis	rare
Kidney and urinary tract disorders	Dysuria	common
	Infection of the urinary tract	common
	Creatinine elevation	common
	Renal insufficiency	rare
	Renal tubular acidosis	rare
General disorders and administrative site conditions	Fatigue	very common
	Worsening of a preexisting fatigue	very common
	Adynamia	very common
	Influenza-like illness	very common
	Rigor	very common
	Hematomas	very common

	Erythema	very common
	Pain at the injection site	very common
	Weight loss	very common
	Pain	very common
	Peripheral edema	very common
	Lethargy	common
	Peripheral swelling	common
	Transfusion reaction	common
	Postoperative bleeding complications	common
	Syncope	common
	Breast pain	common
	Postoperative pain	common

Further details of the side effect profile of 5-azacytidine can be found in the current version of the Investigator´s Brochure (IB). The IB is updated on a regular basis.

7.3.2 Standard cytotoxic therapy (Cytarabine and Daunorubicin)

For information of all known adverse drug reactions please refer to the "Fachinformationsverzeichnis Deutschland" in its latest version.

8 STUDY OUTCOME AND STATISTICAL ANALYSIS

8.1 Power and Sample Size Calculation

A single stage design with fixed sample size was chosen. The accrual time will be 24 months with a follow-up time of 12 months (see 6.4).

Let m_0 and m_1 be the median event free survival times for the control and the 5-azacytidine arm respectively. The Logrank test will be used to test the null hypothesis

$H_0 : m_1/m_0 = 1$

against the two-sided alternative

$H_1: m_1/m_0 \neq 1.$

The allocation ratio was determined to be $n_1/n_0 = 1.0$.

Median event free survival times are supposed to amount to $m_0 = 3$ and $m_1 = 4.5$ in the control and the 5-azacytidine arm respectively (hazard ratio = 0.667). We assume that within 24 months, about 1/3 of recruited study patients will be lost to follow-up, due to alternate post-remission treatment (i.e. bone marrow transplantation), protocol violation or other reasons. The form of the (event free) survival curve as well as the loss curve is assumed to be exponential in both cases.

Resulting from these assumptions, a total of 216 patients (i.e. 108 per group) is required in the controlled part of the study to provide a $1\text{-}\beta=80\%$ power to detect the supposed treatment effect in a two-sided Logrank test with specified $\alpha=0.05$.

Power calculations were performed using SAS version 9.1.3 (SAS Institute Inc., Cary, NC).

8.2 Statistical Analysis Plan

The statistical analysis will be performed according to the intention to treat principle (ITT analysis). This means that every patient included into the study and randomized to one treatment arm will be evaluated in that arm, even if he does not receive study-medication, or if any other violations of the study protocol occur.

- The primary endpoint (median EFS) will be compared between both treatment arms using two-sided stratified Logrank test. This part of the analysis is considered as confirmatory.
- Analyses of secondary endpoints will include, among others, early response rate, morphologic CR-rate, overall survival, leukemia free interval and leukemia free survival.
- In an exploratory approach EFS and OS will be analyzed using stratified Cox-regression. Treatment arm and cytogenetic findings will be included as independent variables. The results of these analyses will be considered as exploratory. No α-adjustment will be performed.

- Patients undergoing allogenic bone marrow transplantation are censored for EFS and OS at the time of bone marrow transplantation.
- In addition to the ITT-analysis a per protocol analysis (PP-analysis) will be performed. This analysis will include only those patients who could be treated with full adherence to the protocol. Besides this, the PP-analysis will parallel the ITT-analysis.

8.3 Number of patients

Under the above assumptions (see 8.1), total accrual to the controlled phase of this trial is planned for 216 patients. The sample size calculation accounts for loss to follow-up in 1/3 of recruited patients (i.e. 72 patients) within 24 months.

8.4 Efficacy

Evaluation of bone marrow aspirate, peripheral blood counts and differentials are used to assess the efficacy of the study medication.

8.4.1 Response criteria

Response criteria are defined according to the Revised Recommendations of the International Working Group for Diagnosis, Standardization of Response Criteria, Treatment Outcomes, and Reporting Standards for Therapeutic Trials in Acute Myeloid Leukemia (14).

Responding Patients:

Morphologic leukemia-free state:

- Bone marrow (aspirate with marrow spicules): < 5% blasts, Absence of Auer rods
- No evidence of persisting leukemia by flow cytometry (Sensitivity: 5%)
- Absence of extramedullary leukemia
- Peripheral blood with no blast cells and either less than 1,000/µl granulocytes and/or less than 100,000/µl platelets

Morphologic Complete Response (CR):

- Platelet count >100,000/µl
- Granulocyte count of >1,000/µl
- Bone marrow (aspirate with marrow spicules): < 5% blasts, Absence of Auer rods
- No evidence of persisting leukemia by flow cytometry (Sensitivity: 5%)
- Absence of extramedullary leukemia
- Transfusion independent stable hemoglobin value

Cytogenetic Complete Response (CRc):

- Platelet count >100,000/µl
- Granulocyte count of >1,000/µl
- Bone marrow (aspirate with marrow spicules): < 5% blasts, Absence of Auer rods

- No evidence of persisting leukemia by flow cytometry (Sensitivity: 5%)
- Absence of extramedullary leukemia
- Transfusion independent stable hemoglobin value
- Normal cytogenetics (based on conventional banded studies and FISH)

Molecular Complete Response (CRm):

- Platelet count >100,000/µl
- Granulocyte count of >1,000/µl
- Bone marrow (aspirate with marrow spicules): < 5% blasts, Absence of Auer rods
- No evidence of persisting leukemia by flow cytometry (Sensitivity: 5%)
- Absence of extramedullary leukemia
- Transfusion independent hemoglobin value
- Normal cytogenetics (based on conventional banded studies and FISH)
- Molecularly negative (no detection of pre-treatment genetic markers with a methodology providing a sensitivity of at least $1:10^3$)

Treatment failure:

Partial Remission (PR):

- Platelet count >100,000/µl
- Granulocyte count of >1,000/µl
- Bone marrow (aspirate with marrow spicules): Decrease of at least 50% in the percentage of blasts to 5% - 25%

Resistant disease:

- Patient survives ≥ 7 days post Chemotherapy (CT); persistent AML in blood or bone marrow

Death in Aplasia:

- Patient survives ≥ 7 days post Chemotherapy (CT); death while cytopenic, with aplastic bone marrow

Indeterminate cause:

- Patients who die < 7 days post CT; Patients who die > 7 days post CT with no PB blasts, but bone marrow examination not performed or not evaluable; Patients who do not complete the first course of therapy

Morphologic relapse:

- Reappearance of blasts post CT in PB or bone marrow

In addition to these response criteria, an early treatment response will be evaluated 6-8 days after the end of the first induction therapy.

Early treatment assessment 6-8 days after end of the first induction therapy:

Good response:

- Bone marrow: < 5% blasts

Bad response:

- Bone marrow: ≥ 5% blasts

8.5 Definition of Study Endpoints

Event free survival (EFS):
Time interval from day 1 of study treatment until treatment failure, relapse from CR, relapse from morphologic leukemia-free state, or death from any cause, whichever occurs first. The time point at which the patient is resistant to therapy or survives induction without a CR or Morphologic leukemia-free state will be noted. For a patient with none of these events before the end of study follow-up, observation of EFS will be censored at the date of his or her last follow-up examination.

Relapse free survival (RFS):

Time interval from the day of documentation of CR until relapse or death from any cause.

Overall survival (OS):
Time interval from day 1 of study treatment to the day of death. For a patient who is not known to have died by the end of follow-up, observation of OS will be censored on the date the patient was last known to be alive.

Complete remission rate (CR rate):
Proportion of patients in complete remission (CR, as defined above) after induction chemotherapy.

Early response rate:
Proportion of patients with a good response (< 5% blasts) in the first treatment assessment 6-8 days after end of the first induction therapy.

9 Quality assurance

9.1 Safety Committee

The Safety Committee of this trial will consist of three members of the scientific community not involved in this trial. The committee will commence regularly (by phone conference) with the Co-ordinating Investigator of the trial and review all serious adverse events (SAE), suspected unexpected severe adverse reactions (SUSARs) and has the right to request any information to be able to detect differences between the treatment arms. The Safety Committee will give recommendations about the continuation of the trial and/or about necessary trial amendments.

9.2 Reference Laboratories

9.2.1 Reference diagnostics for cytomorphology

As stated above, a central review of selected cases will be performed in one of the three diagnostic reference centers.

Dr. U. Schäkel<br>Dr. G. Prange-Krex<br>Medizinische Klinik und Poliklinik I<br>Universitätsklinikum Carl Gustav Carus an der Technischen Universität Dresden<br>Hämatologisches Labor Haus 65a<br>Fetscherstr. 74<br>01307 Dresden<br><br>Phone:0351/458-5627<br>resp. -4251<br>Fax: 0351/458-4367	Dr. med. Utz Krug<br>Prof. Dr. med. C. Müller-Tidow<br>Prof. Dr. med. T. Büchner<br>Universitätsklinikum Münster<br>Medizinische Klinik A<br>Labor für spezielle Hämatologie<br>Albert-Schweitzer-Straße 33<br>48129 Münster<br><br>Phone: 0251/835-2995<br>Fax: 0251/835-2673<br><br>email:<br>Utz.Krug@ukmuenster.de<br><br>AML.Vidaza@uni-muenster.de	Dr. med. B. Steffen<br>Dr. med. U. Brunnberg<br>Dr. med. C. Brandts<br>Universitätsklinikum Frankfurt<br>Medizinische Klinik II<br>Labor für Molekulare Diagnostik<br>Haus 33, UG, Raum 6<br>Theodor-Stern-Kai 7<br>60590 Frankfurt<br><br>Phone: 069/6301-83044<br>Fax: 069/6301-83046

9.3 Monitoring

Before the beginning of the study there will be a central meeting for all investigators from all participating centers (initiation meeting). At this meeting there will be a central training concerning all the relevant details of the study.

During the run-in dose finding phase 100% of the patients will be monitored.

During the course of the study each participating center will be visited for monitoring one to four times. During each of these visits, source data verification will be performed on the basis of a pre-specified sampling plan. The ZKS Muenster will generate this plan. Furthermore, at these visits problematic cases as specified by the principal investigator will be discussed. Study centers can be selected for additional monitoring visits during the course of the study as judged necessary.

At the end of the study there may be a special close out visit for each center.

9.4 Data Management

For data management, an electronic database will be generated at the ZKS Muenster. The database will be equipped with an audit trail that allows for a full follow-up of the history of each item.
Data capture of the study is paper based using pre-specified case report forms (CRF). The completed CRF will be sent to the ZKS as soon as the requested data are available. Double data entry will be done at the ZKS Muenster. Data will be assessed for plausibility. The ZKS will try to resolve possible implausibility by queries to the responsible investigator at the involved center. The investigator has to respond by a signed written statement to be sent to the ZKS Muenster.
The final data set will be handed over to the Institut für Medizinische Informatik und Biomathematik der Universität Münster (IMIB) and to the Co-ordinating Investigator. The biometrical analysis will be done at the IMIB in close cooperation with the Co-ordinating Investigator.

10 INVESTIGATOR’S RESPONSIBILITIES, ETHICAL CONSIDERATIONS, CONFIDENTIALITY, INSURANCE

10.1 Investigator’s responsibilities

The Principal Investigator has more than two years experience in the conductance of clinical trials.

10.1.1 Declaration of Helsinki and GCP compliance

The Investigator undertakes to perform the study in accordance with the Declaration of Helsinki (in its latest version Tokio, 2004) and the ICH Guidelines in Good Clinical Practice as well as with the applicable regulatory requirements.

10.1.2 Protocol adherence

The Investigator must adhere to the protocol as detailed in this document. The Investigator will be responsible for enrolling only those patients who have met protocol eligibility criteria.

10.1.3 Documentation and retention of records

10.1.3.1 Case Report Forms (CRFs)

The Investigator is responsible for maintaining adequate and accurate CRFs which have been designed to record all observations and other data pertinent to the clinical investigation. CRFs should be filled out completely by the Investigator or delegate as stated in the Site Delegation List. All CRFs should be completed in a neat, legible manner to ensure accurate interpretation of the data; a black ball-point pen should be used to ensure the clarity of reproduced copies of all CRFs.

As described in the ICH GCP Guidelines (E6), ‘essential documents’, including CRFs, source documents, consent forms, laboratory test results, and medication inventory records, will be archived by the Investigator according to the current rules and regulations.

10.1.3.2 Recording, access, and retention of Source Data

Source documents are defined as any document where the information is collected during the study procedures for a specific subject. Source documents can be patient’s medical file, appointment books, original laboratory reports, X-rays, Investigators or nurses notes, etc.. Source data to be collected during this study will include, but is not restricted to: patient’s medical file, original laboratory reports, histology, and pathology reports.

10.1.4 Competent local authorities

It is the responsibility of the Investigator to notify the competent local authority about the conduct of this trial before starting recruitment. The Investigator shall inform the competent local authority within 90 days of termination of the clinical trial. Where the clinical trial has been suspended or interrupted

by the sponsor, notification shall take place within 15 days, giving the reasons for suspension or interruption.

10.2 Ethical considerations

10.2.1 Institutional Review Board / Independent Ethics Committee approval

It is the responsibility of the Investigator to provide all requested information about qualification of the respective trial site and trial staff to the sponsor. The sponsor will submit the application to the IRB/IEC.

The trial may only be conducted as approved by the Ethics Committee and the competent authority. Amendments may only be implemented after approval. Additional trial sites may only recruit patients after the sponsor obtained approval for the site.

In compliance with European regulations/ICH-GCP Guidelines, it is required that the Investigator and institution permit authorized representatives of the study central office and the regulatory agency(s) direct access to review the subject's original medical records for verification of study-related procedures and data. Direct access includes examining, analyzing, verifying, and reproducing any records and reports that are important to the evaluation of the study. The Investigator is responsible for giving any requested support for any monitoring, inspection or audit visit. The Investigator has to be available during these visits.

10.2.2 Informed Consent

It is the responsibility of the Investigator to obtain written Informed Consent from patients. Each patient or the patient's legal guardian is requested to sign the Patient Information and Consent Form after the patient has received written information and an explanation of what the study involves (i.e., the objectives, potential benefits and risk, inconveniences and the patient's rights and responsibilities). A copy of the patient Information and signed Consent Form must be given to the patient or the patient's legal guardian.

10.3 Confidentiality

The Investigator must ensure that the patient's encryption is maintained. On the CRFs or other documents submitted to the study central office, subjects should be identified by a subject study number only. Documents that are not for submission to the study central office (e.g., signed informed consent forms) should be kept in strict confidence by the Investigator.

10.4 Insurance

For all patients in this trial, the sponsor has contracted an insurance covering possible damage to the patients at the Gerling-Konzern Allgemeine Versicherungs-AG. The insurance is part of the general insurance agreement between the Universitätsklinikum Münster AöR, Domagkstraße 5, 48149 Münster and the Gerling-Konzern, Policen-Nr.: 70-5630672-8.

Adress of the Insurance Company:

Gerling Vertrieb Deutschland GmbH

Gerling-Konzern
Allgemeine Versicherungs-AG
GIS / Liability
Gereonshof 8
50597 Köln

11 References

1. Büchner T, Hiddemann W, Wormann B, et al. Double induction strategy for acute myeloid leukemia: the effect of high-dose cytarabine with mitoxantrone instead of standard-dose cytarabine with daunorubicin and 6-thioguanine: a randomized trial by the German AML Cooperative Group. Blood 1999;93(12):4116-24.

2. Stone RM, Berg DT, George SL, et al. Postremission therapy in older patients with de novo acute myeloid leukemia: a randomized trial comparing mitoxantrone and intermediate-dose cytarabine with standard-dose cytarabine. Blood 2001;98(3):548-53.

3. Mayer RJ, Davis RB, Schiffer CA, et al. Intensive postremission chemotherapy in adults with acute myeloid leukemia. Cancer and Leukemia Group B. N Engl J Med 1994;331(14):896-903.

4. Krug U, Serve H, Müller-Tidow C, et al. New molecular therapy targets in acute myeloid leukemia. Recent Results Cancer Res. 2007;176:243-62.

5. Gilliland DG, Griffin JD. The roles of FLT3 in hematopoiesis and leukemia. Blood 2002;100(5):1532-42.

6. Thiede C, Steudel C, Mohr B, et al. Analysis of FLT3-activating mutations in 979 patients with acute myelogenous leukemia: association with FAB subtypes and identification of subgroups with poor prognosis. Blood 2002;99(12):4326-35.

7. Müller CI, Rüter B, Koeffler HP, Lübbert M. DNA hypermethylation of myeloid cells, a novel therapeutic target in MDS and AML. Curr Pharm Biotechnol. 2006;7(5):315-21.

8. Glover AB, Leyland-Jones B. Biochemistry of azacitidine: a review. Cancer Treat Rep. 1987;71(10):959-64.

9. Fabre C, Gardin C, Mbida R-M, et al.: Treatment of AML with Azacytidine (AZA): Current Results of the French ATU Program. Blood 2007;110:547a.

10. Avramis VI, Mecum RA, Nyce J, Steele DA, Holcenberg JS: Pharmacodynamic and DNA methylation studies of high-dose 1-beta-D-arabinofuranosyl cytosine before and after in vivo 5-azacytidine treatment in pediatric patients with refractory acute lymphocytic leukemia. Cancer Chemother Pharmacol 1989; 24: 203-10.

11. Borthakur G, Kantarjian H, O'Brien S, et al.: Report of a Phase 1/2 Study of 5-Azacitidine and Cytarabine in Patients with Relapsed, Refractory Acute Myelogenous Leukemia. Blood 2007;110:277a.

12. Kong XB, Tong WP, Chou TC: Induction of deoxycytidine kinase by 5-azacytidine in an HL-60 cell line resistant to arabinosylcytosine. Mol Pharmacol 1991;39:250-7.

13. Levy RJ, Sparano JA, Khan G. Rhabdomyolysis: an unusual complication of cytotoxic chemotherapy. Med Oncol. 1995;12(4):219-22.

14. Cheson BD, Bennett JM, Kopecky KJ, et al. Revised recommendations of the International Working Group for Diagnosis, Standardization of Response Criteria, Treatment Outcomes, and Reporting Standards for Therapeutic Trials in Acute Myeloid Leukemia. J Clin Oncol 2003;21(24):4642-9.

12 Appendices

12.1 Synopsis of the study (in German)

Studientitel	Eine randomisierte, multizentrische Studie zur Erfassung der Effektivität der Gabe von 5-Azacytidin zusätzlich zur Standard-Erstlinientherapie bei Patienten ≥ 61 Jahre mit neudiagnostizierter AML
Klinische Phase	II
Primäres Studienziel	Vergleich des medianen EFS zwischen Studien- und Kontrollarm
Sekundäre Studienziele	• Vgl. des medianen EFS zwischen Pat. mit verschiedenen zytogenetischen und molekularen Risikogruppen[a]<br>• Vgl. des medianen OS zwischen beiden Armen<br>• Vgl. des medianen RFS zwischen beiden Armen<br>• Vgl. des medianen OS zwischen Pat. mit verschiedenen zytogenetischen und molekularen Risikogruppen[1]<br>• Vergleich des Frühansprechens nach dem ersten Induktionskurs zwischen beiden Armen<br>• Vergleich der CR-Rate beider Arme<br>• Vergleich der CR-Rate zwischen Pat. mit verschiedenen zytogenetischen und molekularen Risikogruppen[1]<br>• Vergleich der Rate molekularer Remissionen beider Arme<br>• Vergleich der Toxizität beider Arme<br>• Vergleich des Auftretens minimaler Resterkrankung (MRD) in beiden Armen<br>• Vergleich der Entwicklung/des Vorhandenseins verschiedener Biomarker zwischen beiden Armen<br>• Vergleich der globalen Methylierung / der Methylierung bestimmter Marker-Promotoren zwischen beiden Armen<br>• Evaluation des prädiktiven Wertes einer Änderung des Methylierungsstatus auf das Therapieansprechen im Studienarm<br>[a]Definition der Risikogruppen s. 3.2.2

Studiendesign	Prospektive, kontrollierte, randomisierte, offene multizentrische Phase II Studie mit vorgeschalteter *run-in* Dosisfindungsphase<br><br>Die Patienten erhalten:<br>• Eine modifizierte Induktions- und Konsolidierungstherapie analog zum Intergroup-Protokoll<br>• Arm A: 5-Azacytidin vor den Therapien<br>• Arm A: 5-Azacytidin als Erhaltungstherapie für ein Jahr nach Beginn der Induktionstherapie.<br><br>Induktion 7+3:<br>AraC 100mg/m^2 Tag 1-7<br>Daunorubicin 45mg/m^2 Tag 3-5<br>*Bei Blastenpersistenz am Tag 15 beginnt am Tag 22 ein identischer Zyklus 7+3.*<br><br>Konsolidierung (Hochdosis-AraC):<br>AraC 1g/m^2 (2 x tgl.) Tag 1, 3, 5<br>*Beginn frühestens 1 Woche nach Erreichen der CR, in der Regel nicht früher als 4 Wochen nach Entlassung nach Induktionstherapie. Zweiter Zyklus im Abstand von ≥ 28 Tage nach 1. Zyklus. Bei medizinischer Kontraindikation gegen eine weitere intensive Chemotherapie nach Erreichen der CR wird auf die Konsolidierungstherapie verzichtet; Einzelheiten hierzu s. 5.3.3*<br><br>Die Patienten in Arm A erhalten jeweils an den Tagen (-5) bis (-1) vor den Zytostatika-Gaben 5-Azacytidin; in der Induktionsphase i.d.R. als i.v.-Infusion über 15 bis 30 min., in den Konsolidierungstherapien i.d.R. s.c..<br><br>Die Dosis von Azacytidin vor den Zytostatika-Gaben wird in einer vorgeschalteten *run-in* Dosisfindungsphase ermittelt.<br><br>Erhaltung:<br>Die Patienten im Arm A erhalten nach Regeneration nach dem 2. Konsolidationskurs 5-Azacytidin 75 mg/m^2/Tag s.c. an den Tagen 1-5 eines 4-wöchigen Zyklus bis ein Jahr nach Therapiebeginn. Bei guter Verträglichkeit des 5-Azacytidins ohne Auftreten von Toxizitäten in zwei aufeinander-folgenden Erhaltungstherapiezyklen kann die 5-Azacytidin-Dosis auf 100 mg/m^2/Tag gesteigert werden. Bei schlechter Verträglichkeit wird die Dosis reduziert bzw. ggf. die Erhaltungstherapie beendigt.<br>Die Patienten im Arm B erhalten keine spezifische Erhaltungstherapie, werden aber analog zu den Studienarmpatienten nachuntersucht.
Patientenzahl	216 (108 pro Behandlungsarm) in der kontrollierten Phase
Randomisierung	Die Randomisierung erfolgt über die AML-AZA-Randomisierungs-Hotline des Universitätsklinikums Münster, Tel.: 0251/83-44805.
Einschlusskriterien	• Pat. mit neu diagnostizierter AML (außer APL) nach WHO und/oder FAB-Klassifikation, auch Pat. mit AML aus MDS oder anderer sekundärer AML<br>• Blastenanteil im Knochenmark oder peripheren Blut ≥ 20%. Wenn AML-definierende zytogenetische Aberrationen vorliegen, darf der Blastenanteil auch < 20% sein.<br>• Alter ≥ 61 Jahre<br>• Unterschriebene Einverständniserklärung<br>• Adäquate Empfängnisverhütung

Ausschlusskriterien	• Patienten, die nicht für Standardchemotherapie qualifizieren<br>• Hyperleukozytose (Leukozyten > 20.000/µl) zu Studienbeginn. Diese Patienten sollten mit Hydroxyurea behandelt werden oder Leukapherese (Leukozyten > 100.000/µl) entsprechend dem Standard des jeweiligen Studienzentrums. Sobald Leukozytenzahlen von < 20.000/µl erreicht sind, können diese Patienten in den kontrollierten Teil der Studie eingeschlossen werden, nicht jedoch in den Dosisfindungsteil.<br>• Bekannte ZNS-Manifestation der AML<br>• Herzinsuffizienz NYHA Stadium III oder IV, instabile Angina pectoris (Pat. mit Myokardinfarkt, der länger als 6 Monate zurückliegt, dürfen teilnehmen), schwere ventrikuläre Arrhythmien, welche eine antiarrhythmische Therapie benötigen (mit Ausnahme von β-Blockern und Digitalisglykosiden)<br>• Chronisch eingeschränkte Nierenfunktion (Kreatinin-Clearance < 30ml/min)<br>• Inadäquate Leberfunktion (ALT und / oder AST ≥ 2,5 x ULN), nicht durch AML erklärt.<br>• Gesamtbilirubin ≥ 1,5 x ULN, nicht durch AML erklärt.<br>• Bekannte HIV und/oder Hepatitis-C Infektion<br>• Bestehende oder vorbekannte nicht Leukämie-assoziierte hämorrhagische Diathesen oder Koagulopathien<br>• Bekannte aktive Erkrankungen des ZNS<br>• Unkontrollierte floride Infektion<br>• Zeitgleich bestehende andere bösartige Erkrankung mit einer geschätzten Lebenserwartung von < 2 Jahren<br>• Vorausgegangene Organtransplantation<br>• Bekannte Überempfindlichkeit gegen die Studienmedikation und/oder Chemotherapie sowie ihrer Zusatzstoffe<br>• Vorausgegangene Therapie der AML (ausgenommen Vorphase mit Cytarabin ≤ 2 Tage und Hydroxyurea)<br>• Vorausgegangene Therapie (z.B. eines vorausgegangenen myelodysplastischen Syndroms) mit 5-Azacytidin<br>• Teilnahme an einer anderen Studie in einem Zeitraum von bis zu 4 Wochen vor Studieneinschluss. In diesem Fall Einzelfallentscheidung nach Rücksprache mit dem LKP.<br>• Jegliche Begleitumstände, welche eine Studienteilnahme für den Patienten nicht erstrebenswert erscheinen lassen oder eine protokollgemäße Behandlung behindern

12.2 Performance Status

ECOG		Karnofsky	
Score	Description	Score	Description
0	Fully active, able to carry on all pre-disease performance without restriction.	100	Normal, no complaints, no evidence of disease.
		90	Able to carry on normal activity; minor signs or symptoms of disease.
1	Restricted in physically strenuous activity but ambulatory and able to carry out work of a light or sedentary nature, e.g., light housework, office work.	80	Normal activity with effort; some signs or symptoms of disease.
		70	Cares for self, unable to carry on normal activity or do active work.
2	Ambulatory and capable of all self care but unable to carry out any work activities. Up and about more than 50% of waking hours.	60	Requires occasional assistance, but is able to care for most of his/her needs.
		50	Requires considerable assistance and frequent medical care.
3	Capable of only limited self care, confined to bed or chair more than 50% of waking hours.	40	Disabled, requires special care and assistance.
		30	Severely disabled, hospitalization indicated. Death not imminent.
4	Completely disabled. Cannot carry on any self care. Totally confined to bed or chair.	20	Very sick, hospitalization indicated. Death not imminent.
		10	Moribund, fatal processes progressing rapidly.

12.3 Therapy protocols overview

Therapy Overview

1st Induction	(2nd Induction)	Consolidation 1	Consolidation 2	Maintenance
	only if >5% BM blasts day 15			
7+3 ±Aza	(7+3±Aza)	HiDAC±Aza	HiDAC±Aza	±Aza

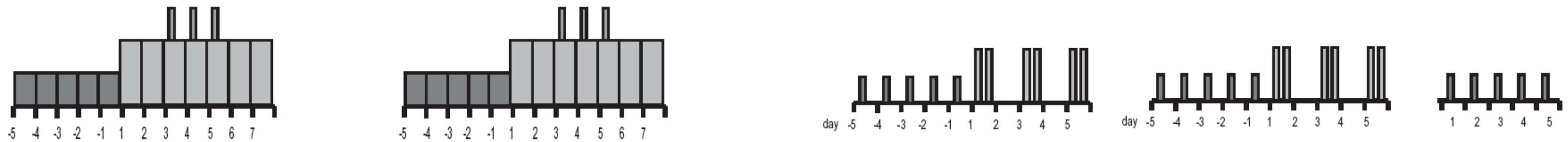

1st Induction	(2nd Induction)	Consolidation 1	Consolidation 2	Maintenance
days 1 to 7:<br>Cytarabine 100 mg/m^2/d 24h i.v.	days 1 to 7 (start day 22)<br>Cytarabine 100 mg/m^2/d 24h i.v.	days 1, 3, 5<br>twice daily<br>Cytarabine<br>1000 mg/m^2 i.v.	days 1, 3, 5<br>twice daily<br>Cytarabine<br>1000 mg/m^2 i.v.	ONLY ARM A<br>days 1 to 5<br>5-Azacytidine<br>75 mg/m^2 s.c.<br>every 4 weeks<br>up to 1 year<br>after induction<br>start<br>Dose modification<br>according to<br>protocol!
days 3 to 5:<br>Daunorubicin 45 mg/m^2 2h i.v.	days 3 to 5 (start day 24)<br>Daunorubicin 45 mg/m^2 2h i.v.			
ONLY ARM A<br>days -5 to -1<br>5-Azacytidine<br>37.5 OR 75 mg/m^2 i.v.	ONLY ARM A<br>days -5 to -1 (start day17)<br>5-Azacytidine<br>37.5 OR 75 mg/m^2 i.v.	ONLY ARM A<br>days -5 to -1<br>5-Azacytidine<br>37.5 OR 75<br>mg/m^2 s.c.	ONLY ARM A<br>days -5 to -1<br>5-Azacytidine<br>37.5 OR 75<br>mg/m^2 s.c.	

ARM A: Azacytidine at days -5 to -1 during induction and consolidation therapy. Plus azacytidine maintenance.
Final concentration of azacytidine (37.5 or 75 mg/m^2) determined in dose finding phase!

Arm A - Induction Therapy (Aza+7+3)

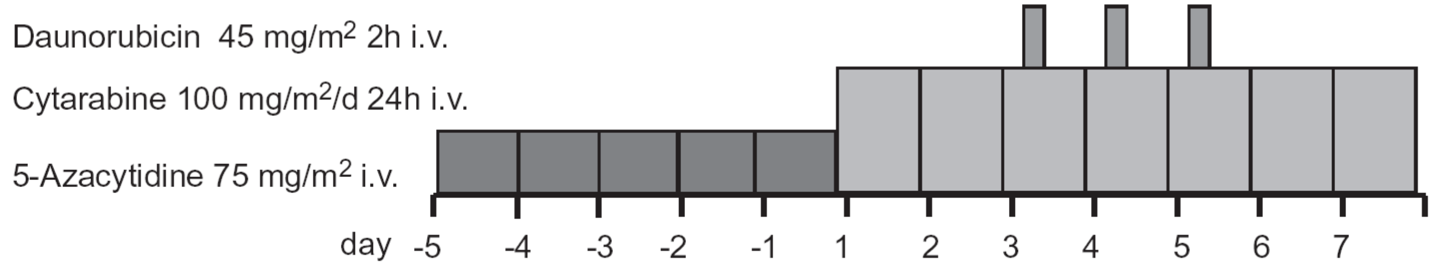

Arm B - Induction Therapy (7+3)

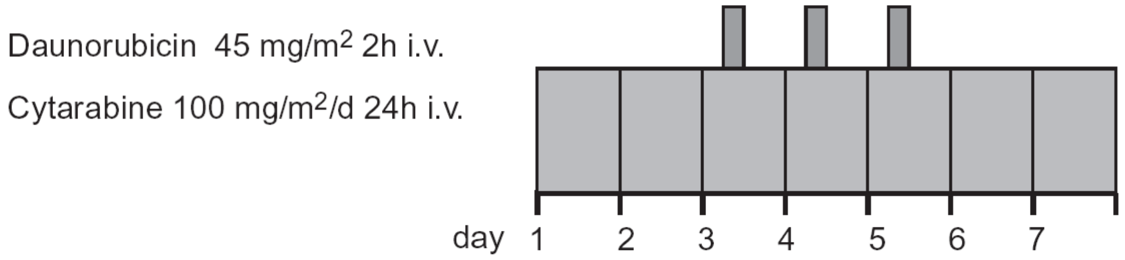

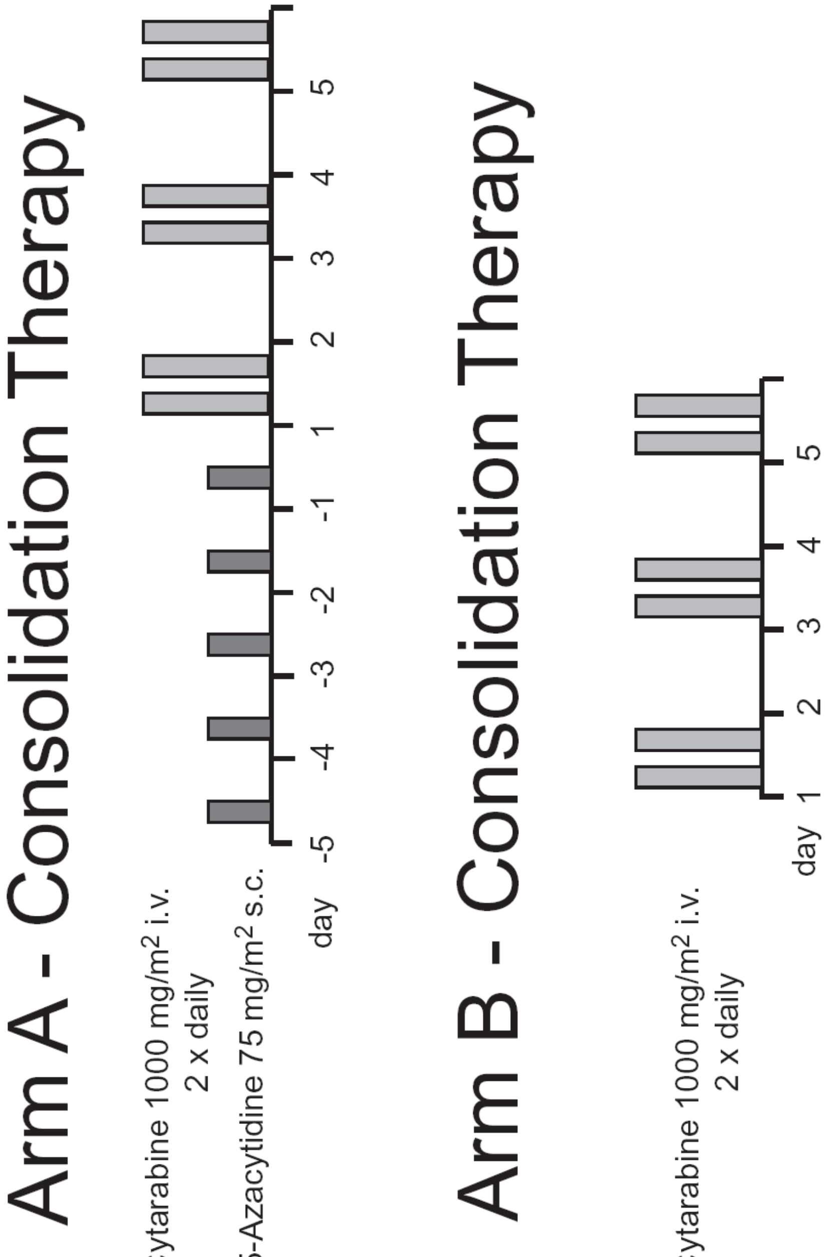
Arm A - Consolidation Therapy
Cytarabine 1000 mg/m2 i.v.
2 x daily
5-Azacytidine 75 mg/m2 s.c.
day
-5
-4
-3
-2
-1
1
2
3
4
5
Arm B - Consolidation Therapy
Cytarabine 1000 mg/m2 i.v.
2 x daily
day
1
2
3
4
5

Arm A - Maintenance Therapy

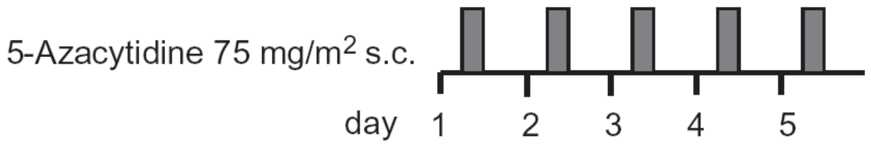

Maintenance therapy starts after regeneration of peripheral blood cell counts after consolidation therapy course 2.
Subcutaneous azacytidine will be administered in 28 day cycles up to one year after start of induction therapy.
Dose modifications as indicated in the study protocol.

Arm B - Follow Up

Patients in Arm B receive similiar supportive care and evaluation as in Arm A but no specific maintenance therapy.
